# MR-Guided Focused Ultrasound-Stimulated Microbubble Radiation Enhancement Treatment for Breast Cancer: Exploratory Imaging Outcomes

**DOI:** 10.3390/cancers18162635

**Published:** 2026-08-15

**Authors:** Anneswa Mukherjee, Laurentius O. Osapoetra, Lakshmanan Sannachi, David Alberico, Maria Lourdes Anzola Pena, Joyce Yip, Gregory J. Czarnota

**Affiliations:** 1 Physical Sciences Platform, Sunnybrook Research Institute, Sunnybrook Health Sciences Centre, Toronto, ON M4N 3M5, Canada; 2 Department of Radiation Oncology, Odette Cancer Centre, Sunnybrook Health Sciences Centre, 2075 Bayview Avenue, Toronto, ON M4N 3M5, Canada; 3 Department of Medical Biophysics, University of Toronto, Toronto, ON M5G 2C4, Canada; 4 Department of Radiation Oncology, University of Toronto, Toronto, ON M4N 3M5, Canada

**Keywords:** microbubble, radiomics, texture analysis, MRI, radiation enhancement, breast cancer, focussed ultrasound

## Abstract

**Background:** A Phase I trial to study the efficacy and safety of MR-guided FUS-MB (focused ultrasound–microbubble) treatment combined with radiotherapy (RT) using a fully electronically MRI-guided steerable focused ultrasound system was performed. The investigation here carried out an exploratory quantitative analysis of images for the first ten participants who were treated with MRgFUS-MB therapy, identifying texture-based MRI features that changed significantly after treatment and indicating probable treatment-related changes in tumour morphology. **Methods and Findings:** Ten patients with stage I-IV breast cancer (Age: 64 ± 10.8) underwent 2 FUS-MB therapies throughout their radiation treatments, as deemed appropriate by a multidisciplinary team. MRI scans were performed prior to treatment and at 1- and 3-month follow-up. The tumours were segmented, and 2D and 3D features, including shape, first-order and texture features, were extracted for original and wavelet-filtered T1-weighted fat-saturated MR images. The overall significance of radiomic features in association with morphological changes in the target tumour over 1-month and 3-month follow-up in comparison to the first treatment day was investigated. **Results:** The five most significant features (*p* < 0.05) were selected using a sample-related *t*-test, out of which most features were wavelet-filtered first-order features. First-order wavelet-filtered robust mean absolute deviation (RMAD) emerged as the most consistent significant feature for both 2D and 3D features with sampled grid spacings of 0.5 mm and 1 mm. The volume of the tumour demonstrated a decline for 80% of patients over 3 months, as measured by the 3D mesh volume of the tumour ROI. There was a significant decline in tumour size and image heterogeneity, indicating plausible changes in the tumour microenvironment possibly related to the treatment. **Conclusions:** Previous preclinical studies and clinical evaluations have demonstrated the efficacy of MR-guided FUS-MB therapy. MRI image-based features from the T1-weighted MR scans over various follow-up times provide potential evidence of changes in tumour tissue heterogeneity that might be treatment-related. MRgFUS MB therapy can possibly evolve as a mode of radiotherapy enhancement in the future, which warrants further controlled validation. The availability of adequate data in the future might possibly lead to the identification of image biomarkers related to treatment response, thus providing better clinical evaluations.

## Simple Summary

Radiation therapy is an essential mode of cancer treatment, and continuous efforts are being made to reduce tissue toxicity while delivering effective treatment. MR-guided focused ultrasound therapy, which uses microbubbles to cavitate and disrupt the tumour vasculature, presumably holds the potential to evolve as an efficient modality for enhancing radiotherapy. The aim of this investigation is to explore the longitudinal radiomic features which exhibit early significant changes that might be associated with changes in tumour morphology. Image analysis was performed for a preliminary population of 10 participants of the MRgFUS MB study to compare tumour morphology at different times. The features showed a significant decline in the values at 1 month for almost 90% of participants irrespective of change in tumour volume, indicating plausible changes in the tumour microenvironment. These findings might form a precursor to the development of robust prognostic markers in the future for treatment response prediction.

## 1. Introduction

Breast cancer in women has emerged as one of the most pervasive forms of cancer, and its global incidence is projected to increase at a staggering rate of 40% by 2040 [1]. Radiation therapy (RT) forms an essential component of oncological management and is a widely used treatment modality for various types of cancer. RT exerts its effects primarily through canonical DNA damage, and higher RT doses per fraction (>8–10 Gy) may enhance tumouricidal efficacy by activating acid sphingomyelinase enzyme (ASMase) in endothelial membranes, leading to ceramide production and eventually causing endothelial cell apoptosis. However, higher doses of radiation pose the risk of damage and toxicity to adjacent healthy tissues. Consequently, the search for an ideal radio sensitizer (selectively targeting tumour cells) continues, with chemotherapy commonly used as a concurrent radio sensitizer in standard clinical practice. Modern RT techniques are increasingly guided by advanced imaging modalities, including ultrasound (US), computed tomography (CT), and magnetic resonance imaging (MRI), to achieve precise tumour targeting and minimize collateral damage to surrounding healthy tissues [2,3,4,5].

However, despite all the belligerent treatment approaches, loco-regional relapse and normal tissue toxicity are the limiting factors in tumour control using RT. One of the most promising strategies to enhance the efficacy of RT while reducing radiation doses involves the use of high-intensity focused ultrasound (HIFU) in addition to RT. Focused ultrasound (FUS) offers high spatial precision, allowing for the selective targeting of tumour tissue while sparing surrounding normal structures [6,7,8].

Recently, a novel combined treatment strategy has been developed, in which intravenously administered microbubbles are stimulated with ultrasound to perturb tumour vascular endothelial cells, thereby enhancing RT effects [9,10,11,12,13,14,15,16]. It was observed in recent studies that under the effect of ultrasound, microbubbles oscillate and cavitate, causing mechanical perturbation of adjacent endothelial cell membranes. The membrane perforations generated by ultrasound stimulated microbubbles (USMB) result in an ASMase-dependent increase in ceramide, thus causing endothelial cell death, synergizing with low doses of radiation. Several preclinical studies demonstrated the feasibility of ultrasound image-guided USMB treatment and radiation enhancement effects in xenograft breast cancer models and more clinically relevant prostate tumour models.

The first Phase I interventional study involving human subjects to study the safety and efficacy of MRI-guided FUS-MB therapy in concurrence with external beam RT was performed with a commercially available MRg-FUS platform (Profound Medical/Philips Sonalleve, Mississauga, ON, Canada/Philips Healthcare, Best, The Netherlands). However, the Sonalleve system employs mechanical translation combined with a phased-array transducer capable of limited electronic steering. Mechanical translation is inherently slower than electronic steering, which can reduce procedural efficiency and introduce motion artifacts in MRI, potentially affecting MR thermometry quality. The technical aspects of different ultrasound systems were investigated and are to be reported elsewhere.

To address these limitations, a fully electronically steerable, MRI-compatible FUS system, Symphony (Arrayus Technologies, Burlington, ON, Canada), was developed, building on the earlier stimulation studies by Ellens and Hynynen [17]. Initially, Symphony was designed for treating uterine fibroids, offering fast, accurate, and motion-free steering capabilities [18]. Recent Phase I human trials have demonstrated the clinical efficacy of the USMB + RT treatment modality, but it would be useful to attempt to identify the image biomarkers that indicate effective tumouricidal effects probably related to treatment. Radiomic analysis involving various imaging modalities such as magnetic resonance imaging (MRI), computed tomography (CT) and qualitative ultrasound (QUS) is a common and useful technique to derive texture features from the acquired images. The concept underlying the process is that both morphological and functional clinical images contain qualitative and quantitative information, which may reflect the underlying pathophysiology of a tissue [19].

The aim of this study was to perform an exploratory longitudinal MRI radiomics analysis of patients treated with MRgFUS-MB plus RT and to identify imaging features associated with radiological changes.

We identified first-order and textural features at 1 and 3 months post-treatment that demonstrated statistically significant changes compared to baseline (day 1). The texture maps were generated to examine the relationship between temporal changes in the most statistically significant features and the gradual morphological evolution of the tumour. In the study reported here, eleven tumours from the first eleven patients with breast carcinoma, primarily with skin or chest wall involvement, with majority of cases presenting distant metastases, were treated with Symphony-aided MR + FUS treatment. The investigation in this paper demonstrates noteworthy tumour size reduction and lowered contrast uptake in most of the cases.

## 2. Materials and Methods

### 2.1. Study Design and Participants

This Phase I single-center, single-arm investigator-initiated interventional study was conducted at Sunnybrook Health Sciences Centre, Toronto, and investigated the feasibility of the MRgFUS treatment method. The Sunnybrook Health Sciences Centre research ethics committee approved the study (REB 5767), and it was registered with clinicaltrials.gov (NCT06633601). We aimed to enroll 30 patients referred for RT at Sunnybrook Health Sciences Centre, and this study constitutes the preliminary analysis of imaging effects after the treatment of 10 patients out of the first 11 patients who were enrolled and treated. Eligibility criteria included patients older than 18 years and a multidisciplinary team of medical, surgical, and radiation oncologists advising RT for stage I–IV breast cancer with a primary or recurrent breast or chest wall tumour in situ. The other factors taken into consideration for inclusion in the study were normal coagulation profile, normal liver and renal function tests (a prerequisite for MB treatment), and weight <140 kg (a requirement for being able to be scanned on the MRI machine). Exclusion criteria included pregnant or lactating women, inability to undergo contrast-enhanced MRI, presence of breast or other metallic implants, bleeding index lesions and presence of fibrotic scar in the US beam path. Factors like history of allergy to microbubbles, history of cardiac disease, uncontrolled hypertension (diastolic BP > 100 mm Hg), history of coagulopathy, use of anticoagulants, estimated glomerular filtration rate <30 mL/min, and Eastern Cooperative Oncology Group (ECOG) performance ≥3 also contributed to exclusion of the patient from the study. The research adhered to good clinical practice guidelines and followed the principles of the Helsinki Declaration. All study participants signed a written consent form before study participation. Clinical study outcomes are reported elsewhere.

### 2.2. Treatment Planning and Procedure:

The radiation treatment and the MRgFUS MB therapy were performed on an outpatient basis. Various imaging modalities were used to outline the tumour target volume for RT (MRI with fusion of volume to CT for dose calculations as part of clinical care) and pre-planning of FUS therapy (MRI). Decisions regarding RT target volumes treated (tumour or whole breast) and radiation dose prescribed were taken by the responsible radiation oncologist and were not influenced by participation in the current study [20]. The radiation therapies were delivered using an image-guided linear accelerator applying forward-planning field-in-field technique (3-dimensional conformal radiotherapy, 3DCRT) or inverse planning intensity-modulated RT.

The MR system consisting of a Philips Ingenia Elition X system (Philips Healthcare, Best, The Netherlands), featuring a 70 cm bore and a magnetic field strength of 3.0 T, was used along with the FUS system Symphony (Arrayus Technologies, Burlington, ON, Canada) for the treatment of Phase I participants.

Most patients included in the study received 5 or 10 fractions of radiation therapy with a total of two MRgFUS-MB treatments performed on days 1 and 5, respectively. On the day of FUS-MB treatment, prior to treatment, a quality control phantom was used to calibrate the alignment between the FUS system focus and the MRI coordinate axes. Then, an intravenous cannula was inserted in the antecubital region of the patient to inject the MRI contrast (Gadavist; Bayer Healthcare Pharmaceuticals, Leverkusen, Germany) and microbubbles (Definity, Lantheus Medical Imaging, Billerca, MA, USA) [20,21]. The patients were positioned prone with the target tumour placed in contact with an ultrasound gel pad measuring 4 cm in height, which helped reduce any irregularities and ensured proper acoustic coupling. Following the calibration and patient positioning, the contrast was injected into the patient, and the treating oncologist utilized the T1-weighted contrast-enhanced MR images from treatment planning to draw the treatment contours based on the size of the target. Each treatment contour can be of arbitrary shape, with approximately 400 points in it, and is considered one treatment cell. Once the treatment compatibility was established, the Definity MB (Lantheus Medical, Billerca, MA, USA) were activated by vigorous agitation for 45 s using a Vialmix unit (Lantheus Medical Imaging, Billerca, MA, USA), and an intravenous dosage of 10 µL/kg followed by 10 mL of saline flush was administered for each treatment cell. Based on previous preclinical optimization, the US exposure time for each treatment point was consistently set to 735 ms over a period of 5 min, and this process was repeated until the treatment was complete for all treatment cells encompassing the tumour target volume. Assuming an attenuation of −1 dB/cm through breast tissue, the input power was adjusted according to the distance between the probe and tumour centre to achieve a negative peak pressure of 570 kPa directed at the tumour centre.

At the conclusion of MRgFUS MB therapy, a contrast-enhanced T1-weighted scan was performed with 0.1 mL/kg of MRI contrast; the patient was monitored for 30 min and then treated with RT within an hour of the previous treatment.

### 2.3. Measurements and Assessments

**Figure 1:**
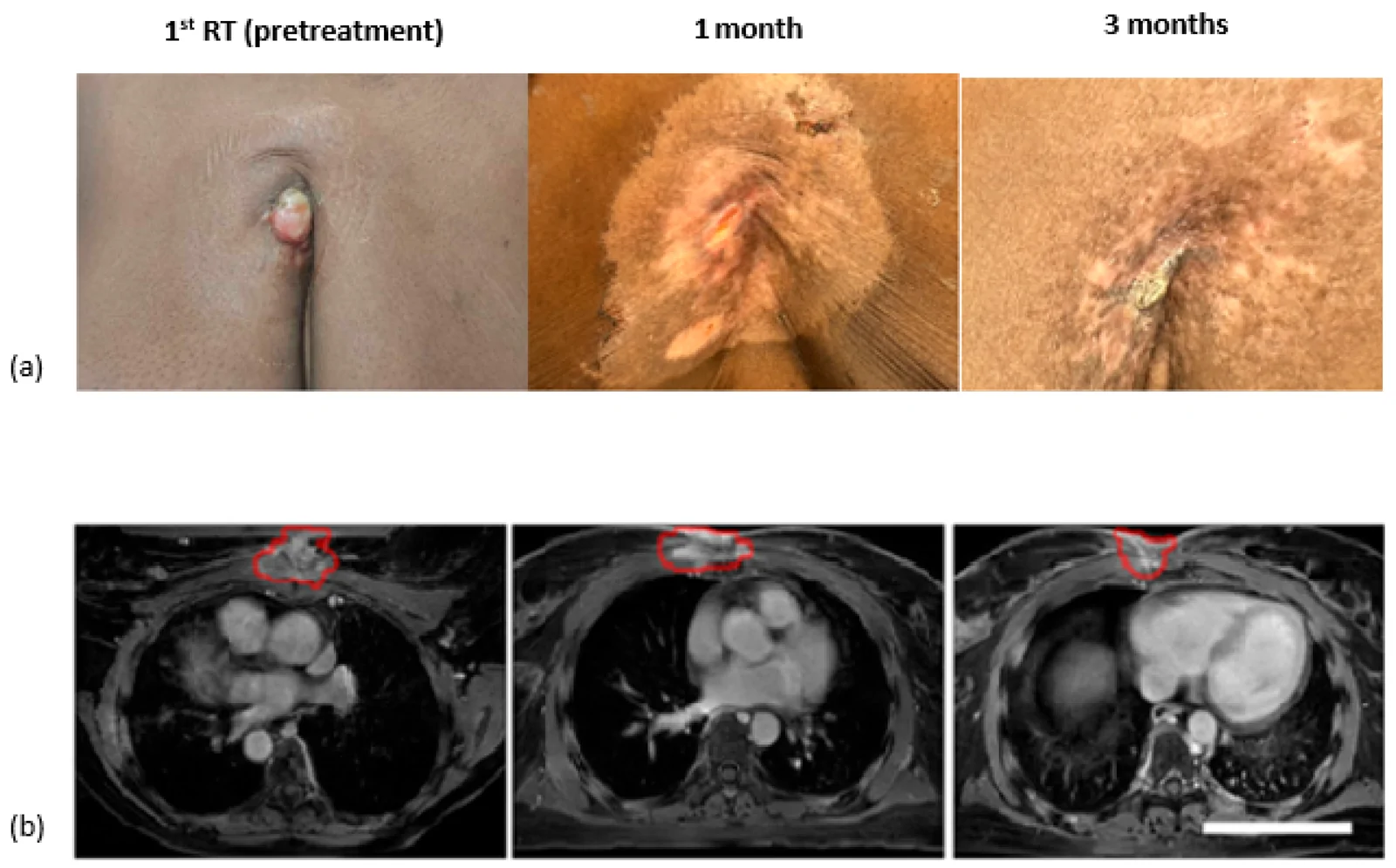
(**a**) Clinical images of the target lesion for a patient on day 1 of MRgFUS MB treatment and 1 month and 3 months after treatment completion. (**b**) CE MRI image of the target lesion for the same patient on the 1st day of MRgFUS MB treatment and after 1 month and 3 months of treatment. The red outline in image (**b**) indicates the ROI contour of the treated tumour. The length bar represents 10 cm.

On the days of the MRgFUS MB treatments, patients were under observation for 1–2 h after the MB injection to ensure that there were no associated complications. The radiation oncologist assessed toxicity effects on the days of MRg FUS MB, 1 week, 1 month and 3 months post-treatment. A complete clinical assessment of toxicity and safety will be produced in a different clinical investigation that will be provided by an experienced radiation oncologist for a larger cohort, at the end of the Phase I study. Safety data for each patient were assessed individually by the principal investigator, one study co-investigator, and a radiation oncologist independent of the study at 2 weeks following FUS-MB and RT treatment. An acute radiation toxicity assessment was performed using the Common Terminology Criteria for Adverse Events (CTCAE) on the last day of RT, and then at 1 month and 3 months after treatment completion. Patients were clinically examined during each follow-up visit until 1 year after treatment to assess any radiation-related late adverse events. Any adverse events were reported to the designated trial monitoring committee within the stipulated regulatory time. The patients who were investigated in this work underwent contrast-enhanced MRI (CE MR) imaging after 1 month and 3 months of study completion, which was analyzed using radiomics to extract the radiological textures and assess radiological changes in the tumour. Figure 1 displays the CE MR image at various time points until 3 months, along with the clinical image during the same period.

### 2.4. Image Feature Analysis

**Figure 2:**
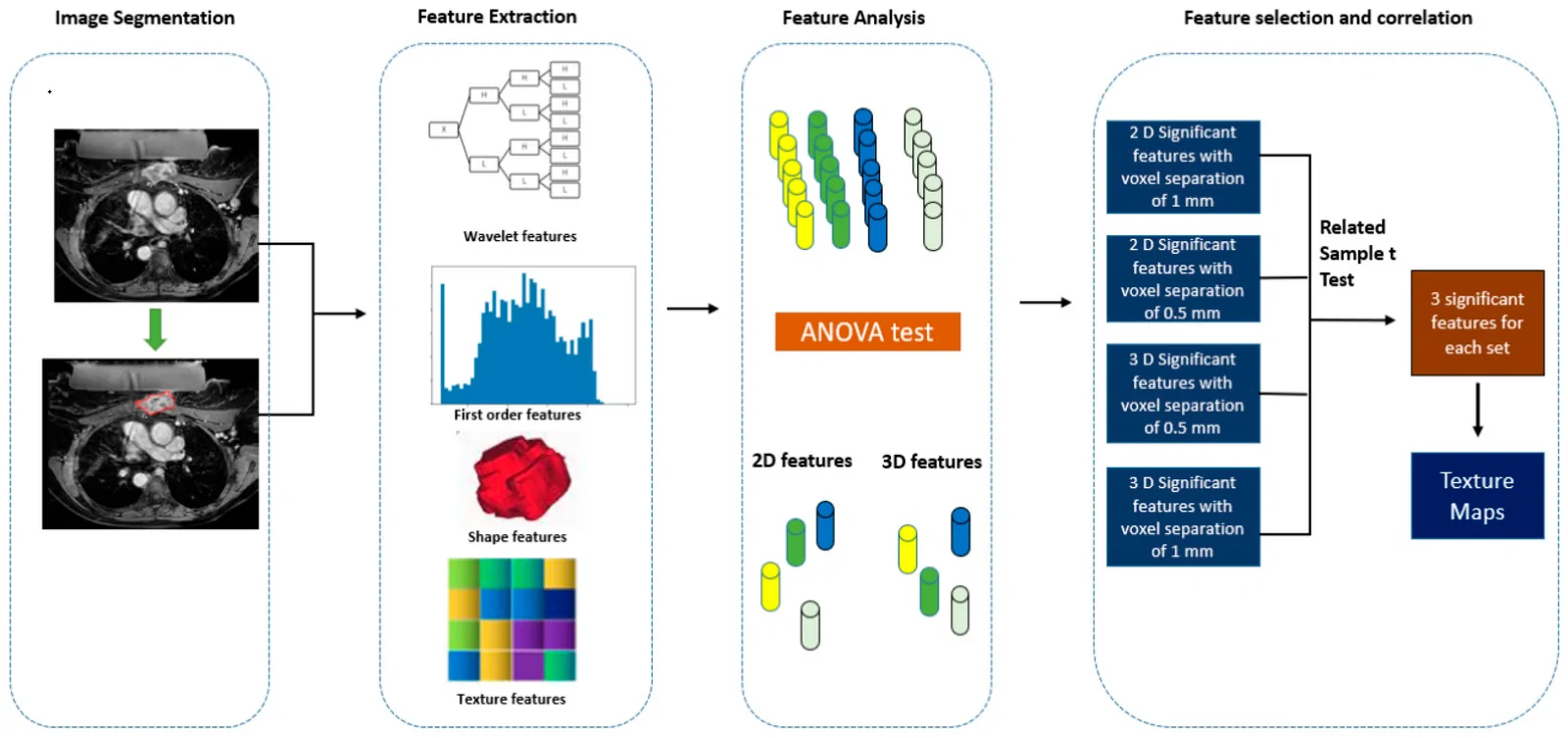
Brief overview of steps involved in radiomic analysis of the MRgFUS MB treatment.

Image analysis is an analytical framework applicable to various target and imaging modalities [22]. There are various steps involved in extracting and classifying data with computational analytical tools. The steps that we followed to analyze the T1-weighted MRI data from the FUS MB treatment and follow-ups at given time points include image acquisition and segmentation, feature extraction, feature selection and investigating the relationship between morphological changes and significant texture features. The overview of steps involved in the radiomic analysis to select significant features is given in Figure 2.

### 2.5. Image Segmentation and Feature Extraction

Handcrafted MRI-based image features were determined from sagittal contrast-enhanced T1-weighted fat-suppressed (T1 FS) MR images of the treated breast tumour.

All patients were scanned with a 3T Philips Ingenia Elition X; the sequences were analyzed from sagittal T1-weighted scans (fat-saturated) as shown in Table 1.

An experienced radiation oncologist (5 years of experience) manually annotated the tumours on the T1 FS-CE images using ITK-Snap software (version 3.x). The reader was not blinded to the time point. The open-source software package PyRadiomics (version 3.1.0) was used to extract these features, enabling a standardized feature extraction process. The standardization process was performed in reference to previous studies performed to standardize MRI scans from multiple scanners with variable parameters to yield robust features [23]. The steps to standardize the data include normalization of pixel intensities, grid resampling and grey-level discretization by fixing bin width as per the Image Biomarker Standardization Initiative (IBSI) [24].

Prior to extracting features, pixel intensities were normalized and scaled to 100. Outliers were removed using a ±3 standard deviation cut-off. Images were then resampled to uniform grids with 0.5 mm and 1.0 mm spacing using the sitkBSpline interpolator in PyRadiomics (version 3.1.0). Specifically, 2D features at 0.5 mm spacing used a 0.5 mm × 0.5 mm grid, and 3D features at 1.0 mm spacing utilized a 1.0 mm × 1.0 mm × 1.0 mm grid.

For feature extraction from the original images, resampled pixels were quantized using a fixed bin width of 10 across all feature types and spatial resolutions. For wavelet-filtered images, bin widths were set to 8 for 2D features at 1 mm resolution, 4 for 2D features at 0.5 mm resolution, 4 for 3D features at 1 mm resolution, and 2 for 3D features at 0.5 mm resolution. Features were extracted using the average of neighbouring distances of 1–5. Wavelet feature extraction followed the default PyRadiomics settings (version 3.1.0), using *coiflets* (Coif1) wavelet decomposition. Decomposition was performed to the first level, producing the ‘LL’, ‘LH’, ‘HL’, and ‘HH’ components, where ‘L’ and ‘H’ denote low- and high-pass filtering.

The extracted features included first-order statistical features (*n* = 18), three-dimensional shape (morphological) features (*n* = 9), and textural features (72). A total of 466 features were extracted for two dimensions and 826 for three dimensions. First-order features characterize the distribution of voxel intensities within the region of interest (ROI), while shape features describe the size and geometry of the ROI [25]. Textural features quantify spatial voxel patterns, capturing properties such as coarseness, contrast, and regularity. These comprised gray-level co-occurrence matrix (GLCM) features (*n* = 24) [26], gray-level run-length matrix (GRLM) features (*n* = 16) [27,28,29,30], gray-level size zone matrix (GLSZM) features (*n* = 16) [31], neighbouring gray tone difference matrix (NGTDM) features (*n* = 5) [32], and gray-level dependence matrix (GLDM) features (*n* = 14) [33]. Textural features were extracted from both original and wavelet-filtered images (listed in Table S1 in Supplementary Material).

### 2.6. Statistical Analysis

A repeated-measures analysis of variance (ANOVA) was utilized to assess differences in feature means across three treatment time points (day 1, 1 month, and 3 months). We utilized the multipletests module from the statsmodels package (version 0.14.5) to adjust uncorrected *p*-values for multiple testing (*n* = 466 for 2D and *n* = 826 for 3D) using the Bonferroni method (Tables S2–S5 in Supplementary Material list all features with corrected *p*-values and effect sizes). Furthermore, post hoc comparisons using related-sample *t*-tests were conducted to identify features whose mean at 1 month and 3 months differed significantly from those at day 1. Statistical significance was set at 0.05, with Bonferroni correction applied to account for multiple comparisons (*n* = 3). The statistical test generated 147 significant features for grid sampling of 0.5 mm and 79 significant features for 1 mm. A total of 175 features and 192 features were significant for three dimensions, with grid spacing of 0.5 mm and 1 mm, respectively. The features with the lowest *p*-values and that were repeatable over most settings were identified as more significant than others. The repeated-measures ANOVA was performed using the AnovaRM class from the statsmodels package (version 0.14.5), and post hoc comparisons were conducted using the ttest_rel function from the scipy package (version 1.13.1).

## 3. Results

Eleven (11) patients who were histologically diagnosed with breast malignancy with simultaneous metastatic disease were treated with FUS MB targeting the primary site in the study; one patient declined follow-ups and was removed from analysis. Patients were referred for the study due to recurrent chest wall disease or due to disease that had become refractory to chemotherapy or systemic therapy. It was found that the median participant age was 64, ranging from 39 to 80 years; the treated tumour sizes ranged from 2 cm to 8 cm, with chest wall and skin involvement in most cases. The patients were treated with a median of 3 mL microbubbles, ranging from 1 to 6 mL, to target the tumour regions initially outlined on the target tumour during treatment planning, followed by a median radiation dose of 35 Gy for five fractions with a dose range of 30 Gy to 40 Gy. Treatment details are presented in Table 2.

None of the patients had any systemic complications or allergic reactions to the MRgFUS MB treatment. The degree of acute toxicity after 3 months of radiation completion was less than grade 2 radiation dermatitis in nine patients and grade 2 dermatitis in one (details will be published in a clinical review after the completion of all participant recruitment and treatments). The tumour volumes were measured by an experienced physician during clinical assessments; the details of the tumour volume at different times, including the percentage volume change at 1 month and 3 months, are provided in Table 3.

During image segmentation and extraction, it was found that around five out of the 10 patients showed a visually significant change in the size of the treated target tumour at the end of 3 months, radiologically. These five patients also demonstrated a more than 80% volume change at 3-month follow-up with respect to the day immediately before treatment, as assessed clinically by an experienced physician specializing in breast cancer and verified by the PI of the study (provided in Table 3).

**Figure 3:**
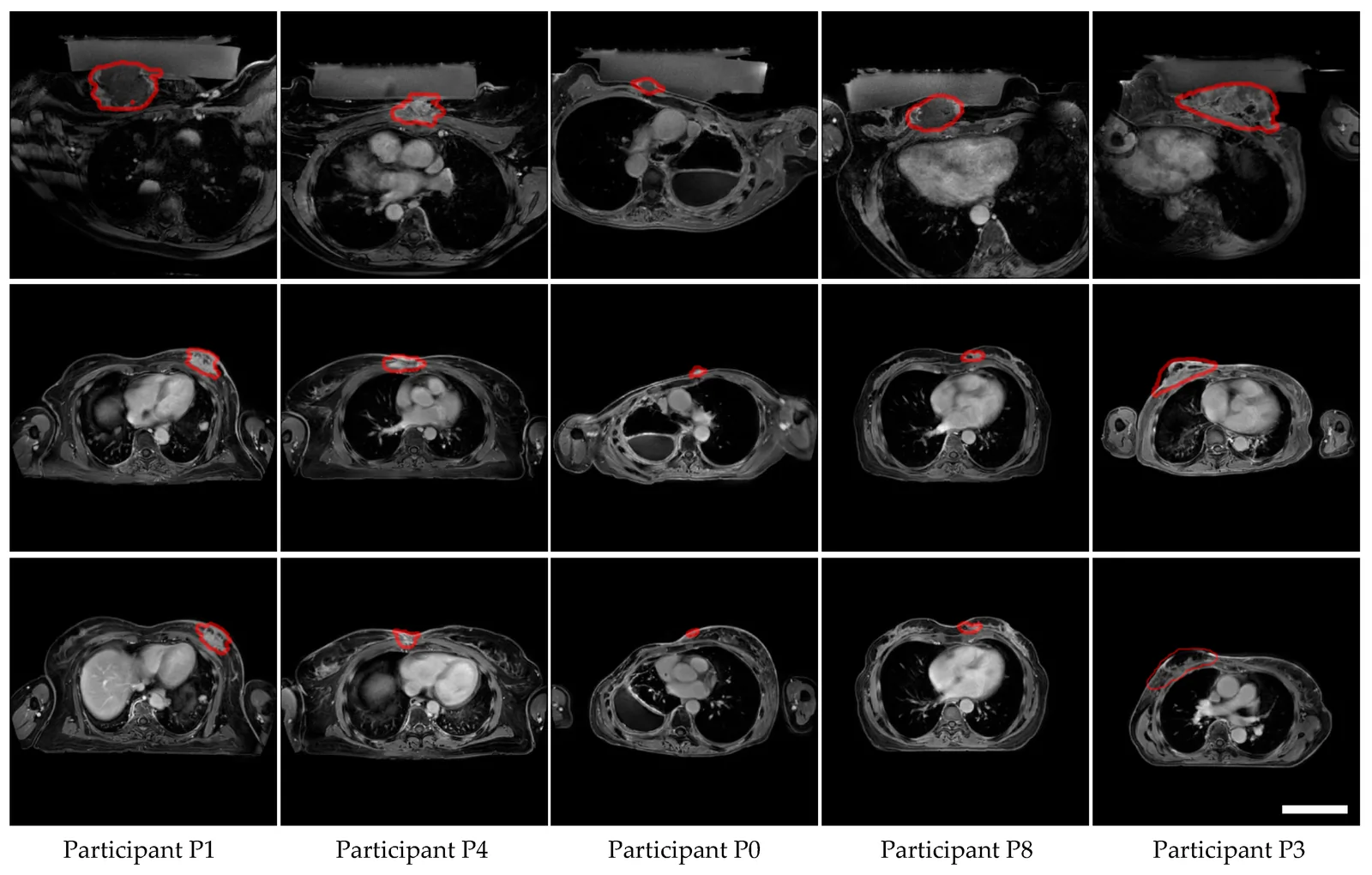
The segmented ROIs for the 5 patients (columns) demonstrate radiological changes in the treated target area over different time points (rows) as follows: on the 1st FUS treatment day (prior to treatment) and 1 month and 3 months after treatment. The red outline in the images indicate the ROI contour of the treated area for individual patients. The scale bar at the bottom right indicates 10 cm.

Figure 3 represents the radiological change in tumour volume with marked ROIs for five individual patient tumours before treatment and at different study follow-up times after treatment completion.

It was obvious from a visual inspection of contrast-enhanced MRI images that targets diminished in size and had diminishment in contrast uptake. Typically, patients demonstrated contrast uptake and then diminishment as early as one week after treatment, which became evident by 3 months after treatment.

Two-dimensional (2D) texture-based image features were determined to objectively characterize changes in targets occurring over three months after treatment.

**Figure 4:**
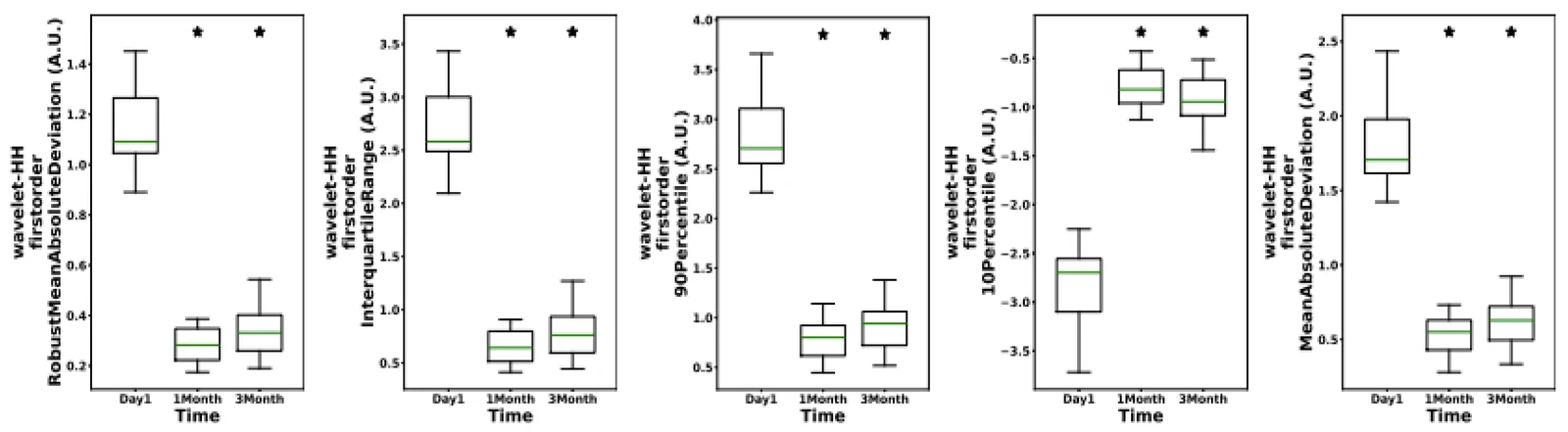
The boxplots display the 5 2D features with 0.5 mm resampled grid spacing that demonstrated significant changes at 1-month and 3-month follow-up in comparison to before treatment (day 1). All 5 significant features (*p* ≤ 0.05) are wavelet-filtered high-pass–high-pass first-order features in the order starting from the left as follows: HH 1st-order robust mean absolute deviation, HH 1st-order interquartile range, HH 1st-order 90th percentile, HH 1st-order 10th percentile and HH first mean absolute deviation. The * at the 1-month time in the boxplots indicates significant change in the set of features at 1 month vs. day 1, and the * at the 3-month time indicates significant change in the set of features at 3 months vs. day 1. The boxplots for the most significant 2D features with grid spacing of 1.0 mm are provided in the Supplementary Material (Figure S1).

**Figure 5:**
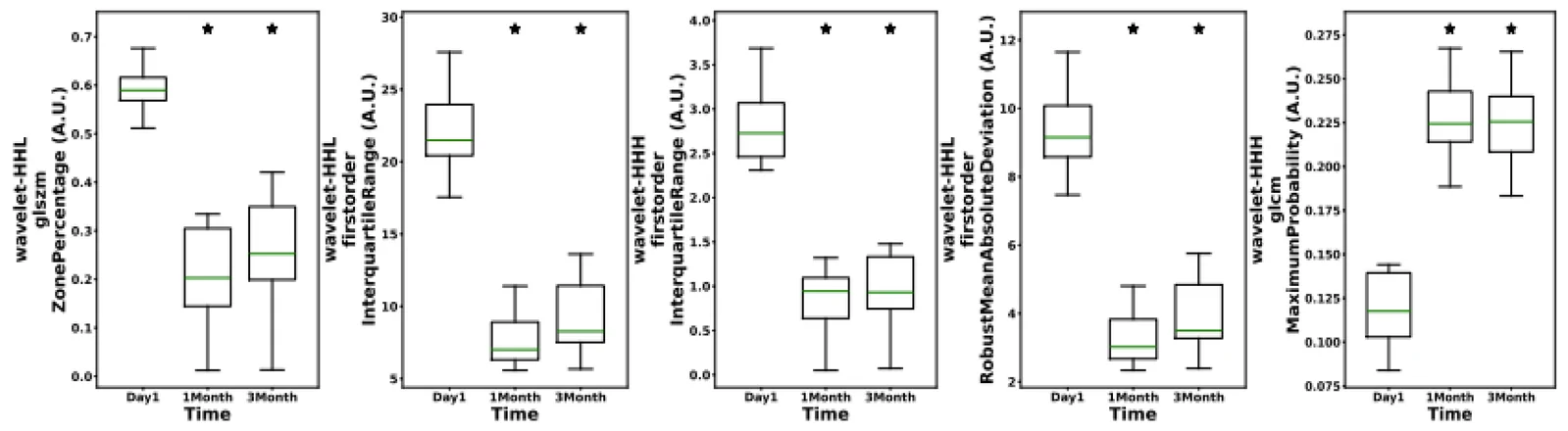
The boxplots display the 5 3D features with 0.5 mm resampled grid spacing that demonstrated significant changes at 1-month and 3-month follow-up in comparison to pre-treatment (day 1). The majority of the 5 significant features (*p* ≤ 0.05) are wavelet-filtered high-pass–high-pass–low-pass features. Starting from the left, these are as follows: HHL GLSZM zone percentage, HHL 1st-order interquartile range, HHH 1st-order interquartile range, HHL 1st-order robust mean absolute deviation and HHH GLCM maximum probability. The * at the 1-month time in the boxplots indicates significant change in the set of features at 1 month vs. day 1, and the * at the 3-month time indicates significant change in the set of features at 3 months vs. day 1. The boxplots for the most significant 3D features with grid spacing of 1.0 mm are provided in the Supplementary Material (Figure S2). GLCM: gray-level co-occurrence matrix, glszm: gray-level size zone matrix.

Based on the related-sample *t*-test performed for the statistically significant set of features, the five most statistically significant features (corrected *p*-values < 0.05) were selected from each set of 2D and 3D features to investigate the correlation of the features with the radiological changes in the size of target volume (which was approximated from the 3D mesh volume extracted from the tumour ROI) over the different time points before and after treatments. The comparison of features was performed between day 1, immediately prior to treatment, and the 1-month follow-up, and between day 1 and the 3-month follow-up. The boxplots in Figure 4 and Figure 5 represent the changes in the five significant 2D and 3D features with a voxel separation of 0.5 mm over three different time points. As visible from the comparison, the difference in feature values for the 1-month follow-up vs. the 3-month follow-up is not quite significant, and no comparison was made.

**Figure 6:**
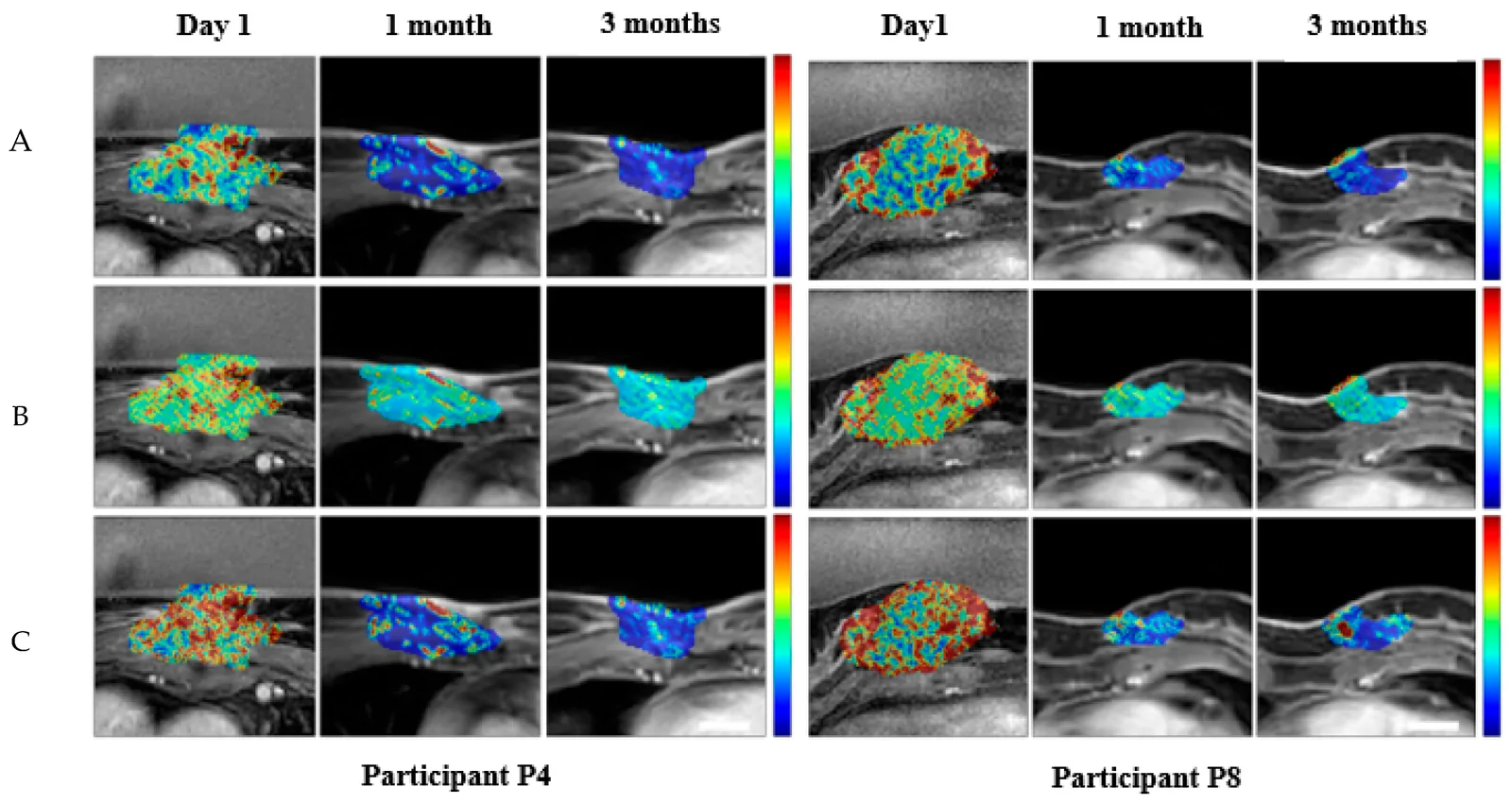
Two-dimensional feature texture maps (0.5 mm spacing) demonstrating 3 significant features for 2 patients on the 1st day of treatment (immediately prior to treatment) and over 1-month and 3-month follow-up in the following order (starting from 1st row): (**A**). HH wavelet 1st-order robust mean absolute deviation (scale range = 0.0–2.00) A.U, (**B**). HH wavelet 1st-order 90th percentile (scale range = −2–5) A.U and (**C**). HH wavelet 1st-order interquartile range (scale range = 0.0–3.0) A.U. The scale is consistent for both patients. The scale bar in the bottom corner represents 2 cm. The texture maps for 2D (1.0 mm spacing) with 3 significant features are provided in the Supplementary Material (Figure S3).

As we intended to sort out the image-based features that might closely relate to morphological changes in the tumour, selecting from the smallest corrected *p*-values (<0.05) from the related-sample *t*-test, the three most significant features were selected for all participants, and the texture maps were plotted. Most of these features were repeatable over the resampled grid spacings of 0.5 mm and 1.0 mm. Representative texture maps for two patients with a visible comparable reduction in size are shown (Figure 6) to visualize the three significant texture features on the day of first treatment and at 1 month and 3 months after treatment. The two representative patients with comparable tumour volume (Table 3) showed significant size reduction, which is visibly apparent, and they received a similar dose of radiation and microbubbles irrespective of the concurrent systemic therapies. The wavelet first-order features with a high-pass filter have been found to be more significant for 2D features, where HH first-order robust mean deviation and HH first-order interquartile range were prevalent for the resampled spacing of 0.5 mm and 1 mm. As can be observed from the texture maps, the variation in intensity is quite high on the day of the first treatment (prior to treatment), but the colours in the map gradually become more consistent (blue colour indicates lower value of deviation), with a few arbitrarily small spikes indicating homogeneity in the tissue, which might be a treatment-related change.

**Figure 7:**
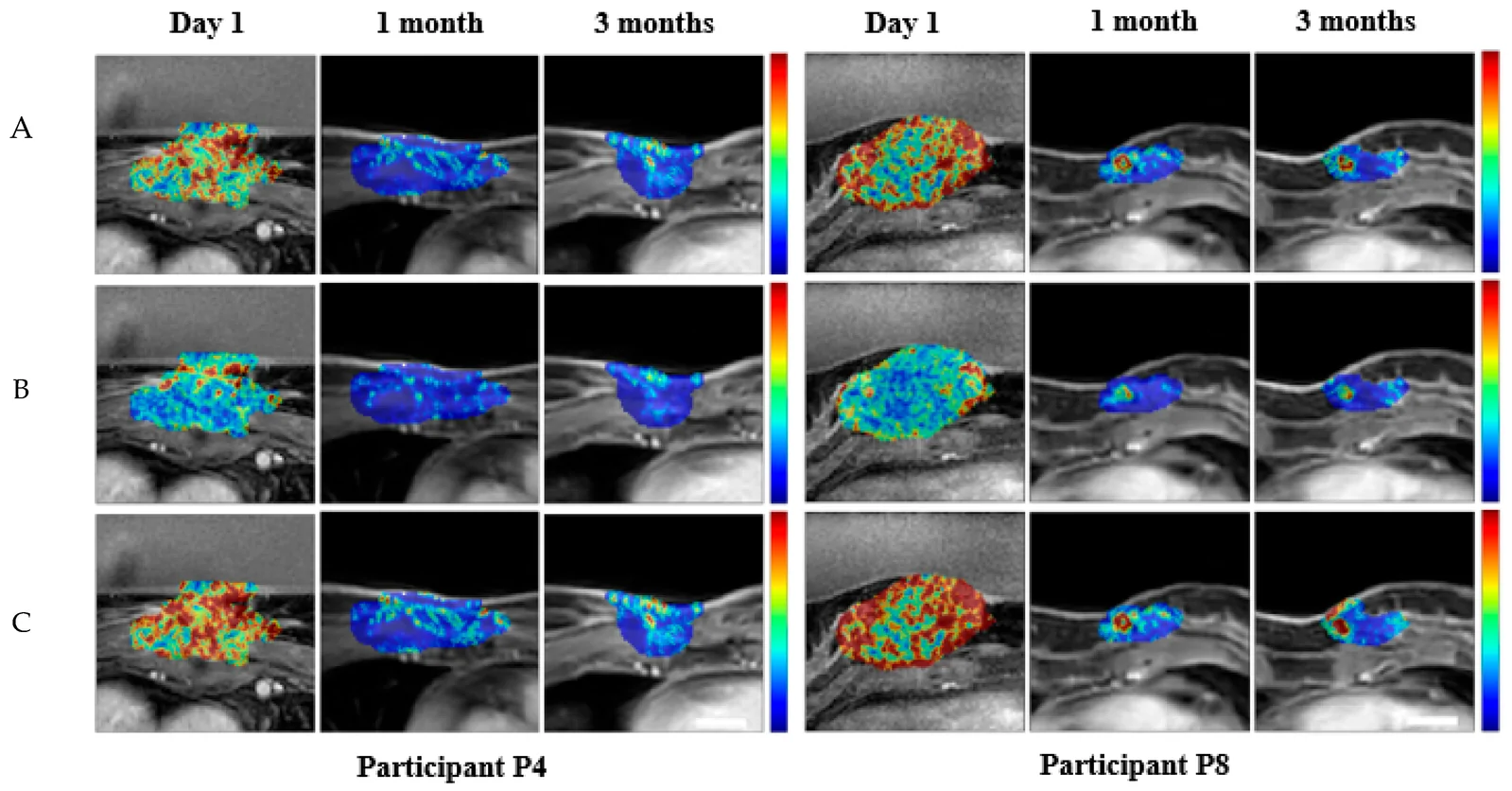
Three-dimensional feature texture maps (0.5 mm spacing) demonstrating 3 significant features for the same set of 2 patients on the 1st day of treatment (prior to treatment) and at 1-month and 3-month follow-up in the following order (starting from 1st row): (**A**) HHL wavelet 1st-order interquartile range (scale range = 0.0–45) A.U, (**B**) HHH wavelet 1st-order interquartile range (scale range = 0.0–5.0) A.U and (**C**) HHL wavelet 1st-order robust mean absolute deviation (scale range = 0.0–18.0) A.U. The colour bar scale range is consistent for both participants in the case of each feature set. The scale bar on the bottom right corner represents 2 cm. The texture maps for 3D (1.0 mm spacing) with 3 significant features are provided in the Supplementary Material (Figure S4).

Features were also assessed for three dimensions, and three-dimensional (3D) feature maps were generated. The changes are more appreciable in the 3D texture map as it is a volumetric representation of the tumour, capturing more discriminative information than a 2D slice. Representative data in Figure 7 illustrate the most significant features for the same set of patients on the first day of treatment and at 1-month and 3-month post-treatment for 3D features, with resampled spacing of 0.5 mm. The set of significant features included first-order wavelet features with three common features for both settings as follows: HHH first-order interquartile range, HHL first-order interquartile range and HHL first-order robust mean absolute deviation. The values for the significant features for the 3 time points are provided in Tables S6 and S7 in the Supplementary Material.

## 4. Discussion

The utilization of quantitative imaging methods has found increasing prevalence in novel personalized medicine approaches in radiotherapy, as radiation responsiveness of tumours is related to some microscopic processes, such as metabolism, hypoxia, perfusion and diffusivity, and several imaging techniques can measure these processes quantitatively [34,35].

Texture-based features computationally determined from imaging data facilitate the objective description of structural heterogeneity of tumour tissues, using quantitative indices derived from statistical and mathematical models applied to the images. Although relatively little data are available on the relationships between the qualitative definition of heterogeneity and image texture features, some studies report indirect relationships [36]. Numerous other publications have investigated the texture features of contrast-enhanced MRI to predict response to various systemic therapies for breast cancer [37,38,39,40] or radiotherapy in non-small cell lung cancer (NSCLC) [41] or in head and neck tumours [42]. In this investigation, the fundamental aim was identifying the significant features that might closely relate to the obvious changes in contrast uptake or volume change of the target tumour over certain time periods. The quantitative analysis of MR images resulted in a considerable reduction in treated tumour volume for majority of participants, and concurrently, several texture features demonstrated decreasing image heterogeneity, indicating tumour response to the combined treatment.

While selecting the statistically most significant feature, which might serve as non-invasive image biomarkers in the future, it was evident that the first-order features showed promising changes for both 2D and 3D settings, with resampled spacing of 0.5 mm and 1 mm. Though both 2D and 3D features have substantial potential, 3D features exhibit superior predictability attributable to comprehensive capture of spatial tumour heterogeneity [43]. Analysis over both dimensions yielded the wavelet-filtered first-order feature that was consistently the most significant across all settings: the wavelet-filtered HHL robust mean absolute deviation (RMAD). In most medical image analysis applications, RMAD is calculated as the mean distance of intensity values from the mean calculated on a subset of the image array (specifically the 10th and 90th percentiles) to reduce the impact of extreme outliers. The RMAD feature relates to the image heterogeneity or significant spatial variation in pixel intensities in the treated ROI. This feature is particularly important in fields such as image processing or analysis, as it can help measure changes in intensity within a specific region. This parameter can quantitatively assess intratumoral heterogeneity by measuring the complexity of intensity or grayscale pixel relationships between different regions. A high value of this feature can be associated with tumour heterogeneity [44]. Even dead necrotic cells have a high RMAD value, but due to differences in cellular and structural organization, the RMAD values for necrosis might not be the same as those of thriving cancer cells. In the same context, fibrotic tissue will have a lower feature value. A lower value of RMAD indicates a homogeneous cell structure, which might be presumably associated with tumour response.

**Figure 8:**
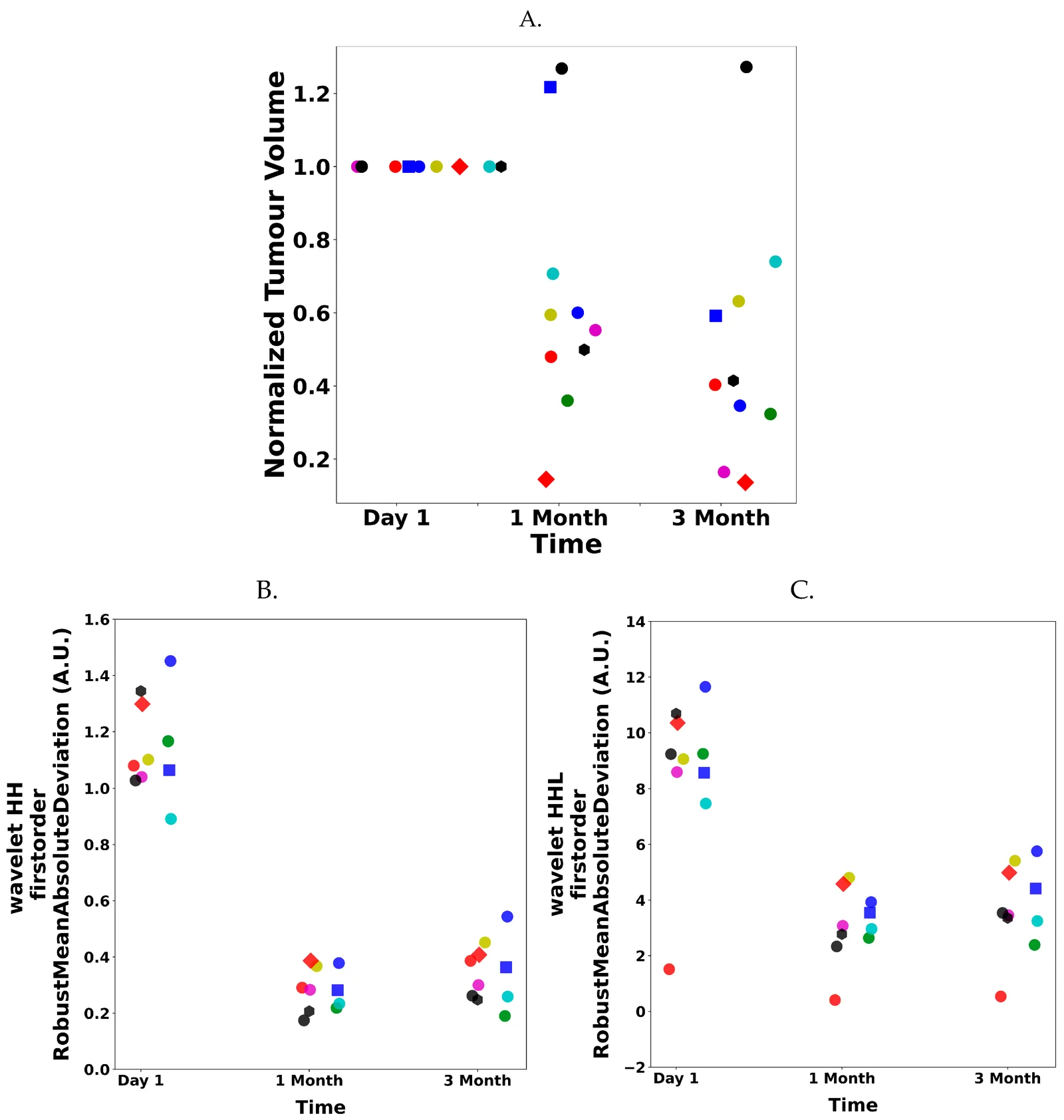
Plots to demonstrate the substantial changes in ROI volume and significant 2D and 3D features for the sample data of ten participants: (**A**). Change in normalized tumour volume on day 1 (immediately prior to treatment) and at 1-month and 3-month follow-up for the patients individually (**B**). Change in 2D wavelet-filtered HH 1st-order robust mean absolute deviation for grid-sampled spacing of 0.5 mm. (**C**). Change in 3D wavelet-filtered HHL 1st-order robust mean absolute deviation for grid-sampled spacing of 0.5 mm. The tumour volume is the extracted 3D mesh volume of the ROI with grid spacing of 0.5 mm. The colour indicates individual patients as follows: (P0 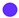 P1 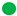 P2 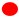 P3 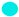 P4 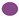 P5 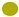 P6 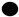 P7 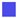 P8 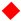 P9 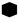).

Even with the modest sample size of 10 patients, it was found that 70% of patients showed considerable change in volume at 3-month follow-up in comparison to the first day of treatment, and it is notable that 60% of participants had tumours with skin involvement or ulceration or both. To measure the volume change over various time periods, the 3D mesh volume data resampled over grid spacings of 0.5 mm and 1 mm, extracted from the segmented ROI of the target tumour, were used. Figure 8A illustrates the changes in normalized 3D mesh volume for resampled spacings of 0.5 mm values of the ROI of treated tumours at three different time points. The treated tumour volume was reduced to almost half for 80% of the treated sample in 1 month after treatment, with a further decrease in volume observed in 70% of the sample patients at 3 months (Figure 8A). The reduction in the ROI mesh volume agrees well with the clinically noted changes in the tumour volume (Table 3). The minor anomalies that appeared in a few cases at 1-month and 3-month follow-up were assumed to be associated with post-radiation inflammation, edema or generous segmentation outlines for the tumour due to manual oversight. The precise biological event relative to the anomaly remains unknown due to the unavailability of pathology and shall be investigated in future. On the other hand, the wavelet-filtered robust mean absolute deviation feature changed significantly over the same time periods, and it is depicted for 2D and 3D settings with a resolution of 0.5 mm in Figure 8B,C. This may be attributed to the fact that at day 1, the tumour bed consisted of a heterogeneous environment of predominantly living tumour cells. As the tumour responded to the combined treatment, this resulted in tumour cell structure change, creating a more homogeneous environment and fibrotic changes over time, thus leading to a reduction in tumour size.

It is quite interesting to note that the target tumours, which demonstrated a volume reduction of almost 60–80% in the first month, consequently had a significant decline in the feature after 1 month, with the RMAD value being one-third of the initial value in 2D settings and half the initial value in 3D settings. It is evident that the value of the wavelet-filtered RMAD feature depreciated considerably in 1 month and then slightly increased after 3 months. This trend is significantly constant for both 2D and 3D feature settings. Presumably, at 1 month and 3 months, the tumoural microstructure consisted of a more homogeneous environment of predominantly responding tumour cells. The lower RMAD value at 1 month indicates lower heterogeneity, and the RMAD of dead cells, fibrosis or vasculature disruption will be much lower than the value observed in the case of thriving tumour tissue (refer to Tables S6 and S7 in Supplementary Material for feature values at given times). The biological reason for the lower RMAD value is difficult to explain in the absence of histopathological evidence; however, it is appreciable that the malignant tumour tissue will have more variation in intensity than other tissue conditions. This might be an indication of positive changes in the tumour microenvironment. It is interesting to observe that irrespective of the volume change of the tumours, the significant feature value reduced over time, which also might indicate changes in the tumour microenvironment without appreciable visual size change in some cases. This provides an opportunity to investigate the relationship between tumour size and significant features in the future with more data.

This work has several limitations. First, the limited amount of sample data and scanner acquisition parameters affect statistical power, thus limiting the generalizability of the results. Extensive standardization techniques were used to abate the probability of false positive findings, and the significant intensity features in our investigation have been found to be robust as per the standardized features list suggested by IBSI after consensus and rigorous validation. It is also intended to expand the work once the clinical study has completed its treatment of patients. Expanding the cohort will facilitate work to build on current findings and perform more robust validation strategies. Second, the reliance on manual ROI annotations will introduce intra- and inter-observer variabilities that might affect the robustness of the analysis. Further studies will aim to incorporate automated ROI delineation utilizing deep neural network architectures.

Another important factor that might be stratified in future investigations is the clinical heterogeneity of the patient cohort. Diverse patient populations might dilute distinct responses, but despite the heterogeneous nature, the imaging outcomes aligned well with the clinical assessment of volume. Moreover, we aimed to explore image features that might have changed significantly for the participants over time rather than making response predictions based on the image markers. Implementing deep learning models and possibly classifying patients based on systemic therapies, disease stage or molecular status might provide sufficient information to plan personalized treatment strategies, improving clinical decision-making and treatment management.

It can be presumably hypothesized from the texture analysis and previous preclinical and clinical research that MR-guided FUS provides decent disease response rates when used synergistically with radiation therapy through the conversion of the tumour target to an avascular structure.

## 5. Conclusions

In summary, it can be concluded from preliminary analysis of sample data that the longitudinal MR images demonstrated exploratory changes in tumour volume and image heterogeneity after MRgFUS MB + RT treatment. The findings suggest that MRI-based radiomic features may help characterize treatment-related imaging changes, but larger controlled studies with biological or functional imaging validation are required to determine whether these features can serve as reliable biomarkers of treatment response. The application of MR-guided microbubbles supra-additively with radiation appears promising. The implications of factors like molecular status and systemic agents that can have possible implications on tumour response are not addressed in this work and will be accounted for as stratification factors in future work. The trend in ROI volume and changes in statistically significant features indicate plausible changes in tumour tissue physiology which might be related to the treatment. The gradual decline in tumour tissue complexity was visually notable in the texture maps, and with further research, the image markers can be identified, which will play a crucial role in patient classification, prediction of treatment response, and prognosis determination. The purpose of future investigation shall be the inclusion of larger groups of participants and constructing robust prediction models using machine learning or deep learning that will effectively contribute to better clinical evaluations and potentially provide improved therapeutic outcomes.

## Figures and Tables

**Table 1:** MRI acquisition parameters for longitudinal post-contrast T1-weighted fat-saturated scans.

Parameters	Day 1 (Prior to Treatment)	Follow Ups (1 Month and 3 Months)
Repetition Time (TR) (ms)	5.2 ms	4.9 ms
Echo Time (TE) (ms)	0	0
Flip Angle (degree)	10	10
Slice Thickness (mm)	3	3
Matrix	384 × 384	448 × 448
Field of View (FOV) mm	100	100
Mode	3D	3D

**Table 2:** Clinical features and MRgFUS MB + RT treatment details of analyzed patient data. The colour-coded pointers are identifiers for the patients. All the participants received a total of 2 MRgFUS MB therapy sessions during their radiation.

Patient	Age	Disease Type	Target Tumour Size and Site	Distant Metastases	Stage T of Cancer	Molecular Status	Microbubble Dose (mL) per Treatment	Concurrent Systemic Therapy	RT Dose/(Total Fractions)	Skin/Chest Wall Involvement
**P0** 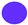	62	Papillary carcinoma. New progression	Left chest wall ~2 cm	Pleura	T4	ER+, PR+, HER2+	1	Herceptin	35 Gy/#5	Skin involvement.
**P1** 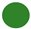	80	Primary progression	Left breast 6.6 cm × 7.3 cm × 7.5 cm	Mediastinum and Lung	T4	ER+, PR+ (<10%), HER2−	6	Letrozole	30 Gy/#5	Skin involvement with ulceration.
**P2** 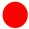	76	IDC primary progression	Right breast mass ~3 cm × 2.9 cm	Bone and cervical spine	T2	ER+, PR+, HER2+	1	Trastuzumab + Pertuzumab	35 Gy/#5	None.
**P3** 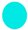	72	IDC primary progression	Right breast ~8 cm	Spine and bones	T4	ER+, PR+, HER2−	5	Letrozole/Enhertu	35 Gy/#5	Both. Ulceration was present as well.
**P4** 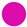	54	IDC, recurrence	Left sternal mass ~3 cm	None	T1	ER+, PR-, HER2−	3	Capecitabine	30 Gy/#5	Chest wall involvement.
**P5** 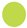	65	IDC primary progression	Right breast ~3–4 cm retroareola	Lung	T4	ER−, PR−, HER2+	2	Pertuzumab after MRgFUS MB	40 Gy/#5	Skin involvement.
**P6** 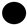	68	Metastatic breast carcinoma, primary progression	Right breast ~2.5 cm	Liver	T2	ER−, PR−, HER2+	1	Trastuzumab + Pertuzumab	30 Gy/#5	None.
**P7** 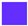	39	IDC primary progression	Left breast mass 6 cm	Pleural effusion	T4	ER+, PR+, HER2-	3	None	40 Gy/#5	Both. Ulceration was present as well.
**P8** 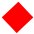	57	IDC primary progression	Left breast mass 3.6 cm × 4.0 cm × 4.8 cm	Liver	T2	ER−, PR−, HER2−	3	Gemicitabine + Caboplatine	30 Gy/#5	None.
**P9** 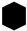	67	IDC primary inflammatory	Left breast side 4 cm × 4 cm	Lung	T3	ER+ (<1%), PR+ (1%), HER2−	4	Gemicitabine + Caboplatine + pebrozulimab	35 Gy/#5	Skin involvement.

**Table 3:** Tumour volume and volume percentage change at different time points for individual participants measured after clinical assessment.

Participant	Contrast Enhanced Tumour Volumes at Various Time Points			Percentage Volume Change at 1-Month and 3-Month w.r.t Day 1	
	Day 1	1 Month	3 Months	Day 1 vs. 1 Month	Day 1 vs. 3 Months
P0	4.79 cc	1.02 cc	<0.01 cc	79%	~100%
P1	120.60 cc	20.13 cc	3.20 cc	83%	97%
P2	54.90 cc	47.80 cc	45.06 cc	13%	18%
P3	375 cc	44 cc	10.54 cc	88%	97%
P4	69.50 cc	36.67 cc	9.60 cc	47%	86%
P5	9.28 cc	4.47 cc	4.77 cc	52%	49%
P6	6.48 cc	3.09 cc	3.33 cc	52%	49%
P7	60.97 cc	28.91 cc	18.22 cc	53%	70%
P8	72.43 cc	5.52 cc	3.37 cc	92%	95%
P9	279 cc	190.5 cc	96.36 cc	32%	65%

## Data Availability

Data are stored in an institutional repository and will be made available on request to the corresponding author following institutional ethics committee protocols.

## Supplementary Materials

The following supporting information can be downloaded at: https://www.mdpi.com/article/10.3390/cancers18162635/s1. Figure S1: The boxplots display the five 2D features with 1.0 mm resampled grid spacing, that demonstrated significant changes in 1 month and 3 months follow-up in comparison to before treatment (Day 1); Figure S2: The boxplots display the five 3D features with 1 mm resampled grid spacing, that demonstrated significant changes in 1 month and 3 months follow-up in comparison to pre- treatment (Day 1); Figure S3: Two dimensional feature texture maps (1.0 mm spacing) demonstrating 3 significant features for 2 patients on 1st day of treatment (immediately prior to treatment) and over 1 month and 3 months follow-up in the following order (starting from 1st row): HH wavelet glszm Zone Percentage (scale range = 0.0–1.4) A.U, HH wavelet glrlm Run length non-uniformity (scale range= 0.0–1.4) A.U and HH wavelet 1st order Interquartile Range (scale range = 0.0–20) A.U.; Figure S4: Three dimensional feature texture maps (1.0 mm spacing) demonstrating 3 significant features for the same set of 2 patients on 1st day of treatment (prior to treatment) and over 1 month and 3 months follow-up in the following order (starting from 1st row): A. HHL wavelet 1st order Interquartile Range (scale range= 0.0–45) A.U, B. HHH wavelet 1st order Interquartile Range (scale range = 0.0–5.0) A.U and C. HHL wavelet 1st order Robust mean absolute deviation (scale range = 0.0–18.0) A.U.; Table S1: Radiomics Features; Table S2: The 2D features with raw *p*-value, corrected *p*-value and effect sizes. (grid separation of 0.5 mm); Table S3: The 3D features with raw *p*-value, corrected *p*-value and effect sizes. (grid separation of 0.5 mm); Table S4: The 2D features with raw *p*-value, corrected *p*-value and effect sizes. (grid separation of 1.0 mm); Table S5: The 3D features with raw *p*-value, corrected *p*-value and effect sizes. (grid separation of 1.0 mm); Table S6: The 3 significant features (2D) for both resampled grid distance of 0.5 mm and 1.0 mm; Table S7. The 3 significant features (3D) for both resampled grid distance of 0.5 mm and 1.0 mm.
