# Supplementary material for: MR-Guided Focused Ultrasound-Stimulated Microbubble Radiation Enhancement Treatment for Breast Cancer: Exploratory Imaging Outcomes
Source: Cancers (Basel). 2026 Aug 15;18(16):2635. doi: 10.3390/cancers18162635 (PMC13510511; doi:10.3390/cancers18162635)
Supplement: Supplementary file 1 [file cancers-18-02635-s001.zip › cancers-4412152-supplementary.pdf]

Supplementary Material

Table S1. Radiomics Features.

First-Order Statistical Features (*n* = 18)	{ 10th Percentile, 90th Percentile, Energy, Entropy, Interquartile Range, Kurtosis, Maximum, Mean Absolute Deviation (MAD), Mean, Median, Minimum, Range, Robust Mean Absolute Deviation (rMAD), Root Mean Squared (RMS), Skewness, Total Energy, Uniformity, Variance }
GLCM (*n* = 24)	{ Autocorrelation, Cluster Prominence, Cluster Shade, Cluster Tendency, Contrast, Correlation, Difference Average, Difference Entropy, Difference Variance, Inverse Difference (ID), Inverse Difference Moment (IDM), Inverse Difference Moment Normalized (IDMN), Inverse Difference Normalized (IDN), Informational Measure of Correlation (IMC) 1, Informational Measure of Correlation (IMC) 2, Inverse Variance, Joint Average, Joint Energy, Joint Entropy, Maximal Correlation Coefficient (MCC), Maximum Probability, Sum Average, Sum Entropy, Sum Squares }
GRLM (*n* = 16)	{ Gray Level NonUniformity, Gray Level NonUniformity Normalized, Gray Level Variance, High Gray Level Run Emphasis, Long Run Emphasis, Long Run High Gray Level Emphasis, Long Run Low Gray Level Emphasis, Low Gray Level Run Emphasis, Run Entropy, Run Length NonUniformity, Run Length NonUniformity Normalized, Run Percentage, Run Variance, Short Run Emphasis, Short Run High Gray Level Emphasis, Short Run Low Gray Level Emphasis }
GLSZM (*n* = 16)	{ Gray Level NonUniformity, Gray Level NonUniformity Normalized, Gray Level Variance, High Gray Level Zone Emphasis, Large Area Emphasis, Large Area High Gray Level Emphasis, Large Area Low Gray Level Emphasis, Low Gray Level Zone Emphasis, Size Zone NonUniformity, Size Zone NonUniformity Normalized, Small Area Emphasis, Small Area High Gray Level Emphasis, Small Area Low Gray Level Emphasis, Zone Entropy, Zone Percentage, Zone Variance }
GLDM (*n* = 14)	{ Dependence Entropy, Dependence NonUniformity, Dependence NonUniformity Normalized, Dependence Variance, Gray Level NonUniformity, Gray Level Variance, High Gray Level Emphasis, Large Dependence Emphasis, Large Dependence High Gray Level Emphasis, Large Dependence Low Gray Level Emphasis, Low Gray Level Emphasis, Small Dependence Emphasis, Small Dependence High Gray Level Emphasis, Small Dependence Low Gray Level Emphasis
NGTDM (*n* = 5)	{ Busyness, Coarseness, Complexity, Contrast, Strength }

GLCM : Gray level co-occurrence matrix

GRLM : Gray level run-length matrix

GLSZM : Gray level size-zone matrix

GLDM : Gray level dependence matrix

NGTDM : Neighbouring gray tone difference matrix

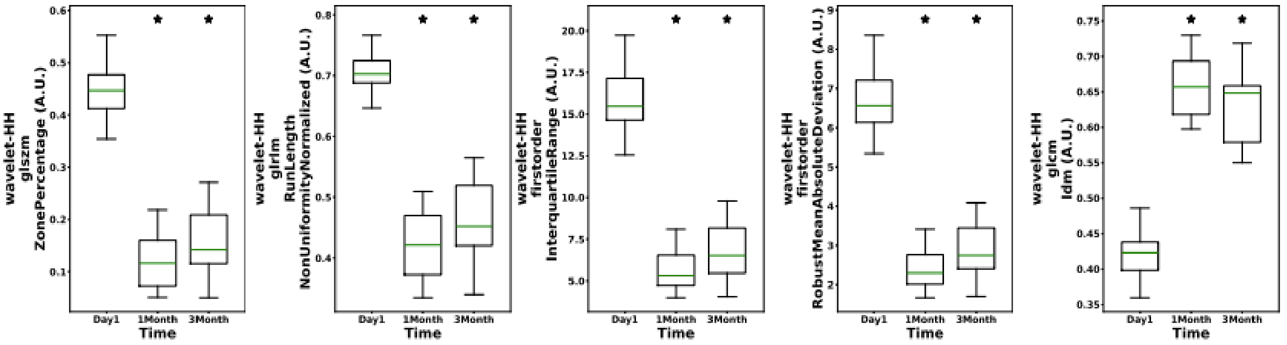

Figure S1. The boxplots display the five 2D features with 1.0 mm resampled grid spacing, that demonstrated significant changes in 1 month and 3 months follow-up in comparison to before treatment (Day 1). All 5 significant features (p≤ 0.05) are wavelet filtered features in the order starting from the left as follows: HH glszm Zone Percentage, HH glrlm Run length Non-Uniformity Normalized, HH 1st order Inter quartile Range, HH 1st order Robust Mean Absolute Deviation and HH 1st glcm Idm. The * at 1month and 3 months indicate significant change in the feature at 1 month vs day 1 of treatment and 3 months vs day 1 of treatment respectively.

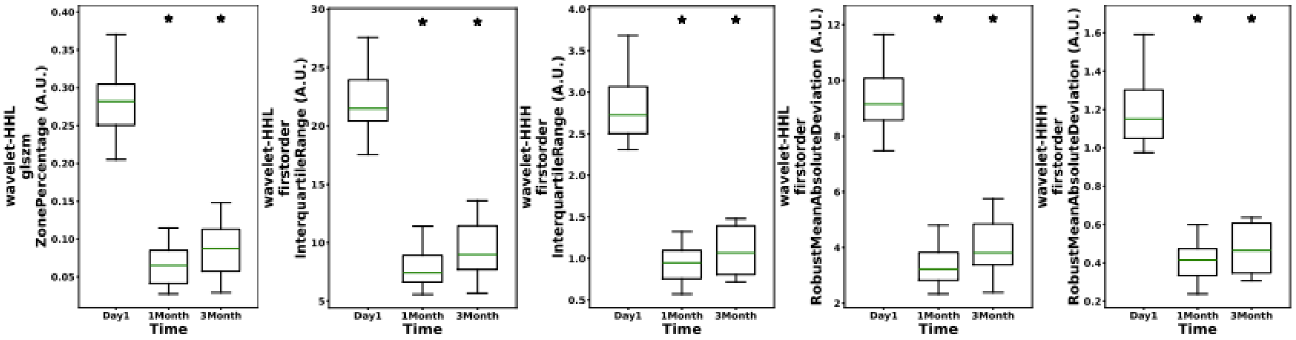

Figure S2. The boxplots display the five 3D features with 1 mm resampled grid spacing, that demonstrated significant changes in 1 month and 3 months follow-up in comparison to pre- treatment (Day 1). Majority of the 5 significant features (p≤ 0.05) are wavelet filtered high pass-high pass-low pass features in the order starting from the left as follows: HHL glszm Zone Percentage, HHL 1st order Inter quartile Range, HHH 1st order Inter quartile Range, HHL 1st order Robust Mean Absolute Deviation and HHH 1st order Robust Mean Absolute Deviation. The * at 1month and 3 months indicate significant change in the feature at 1 month vs day 1 of treatment and 3 months vs day 1 of treatment respectively.

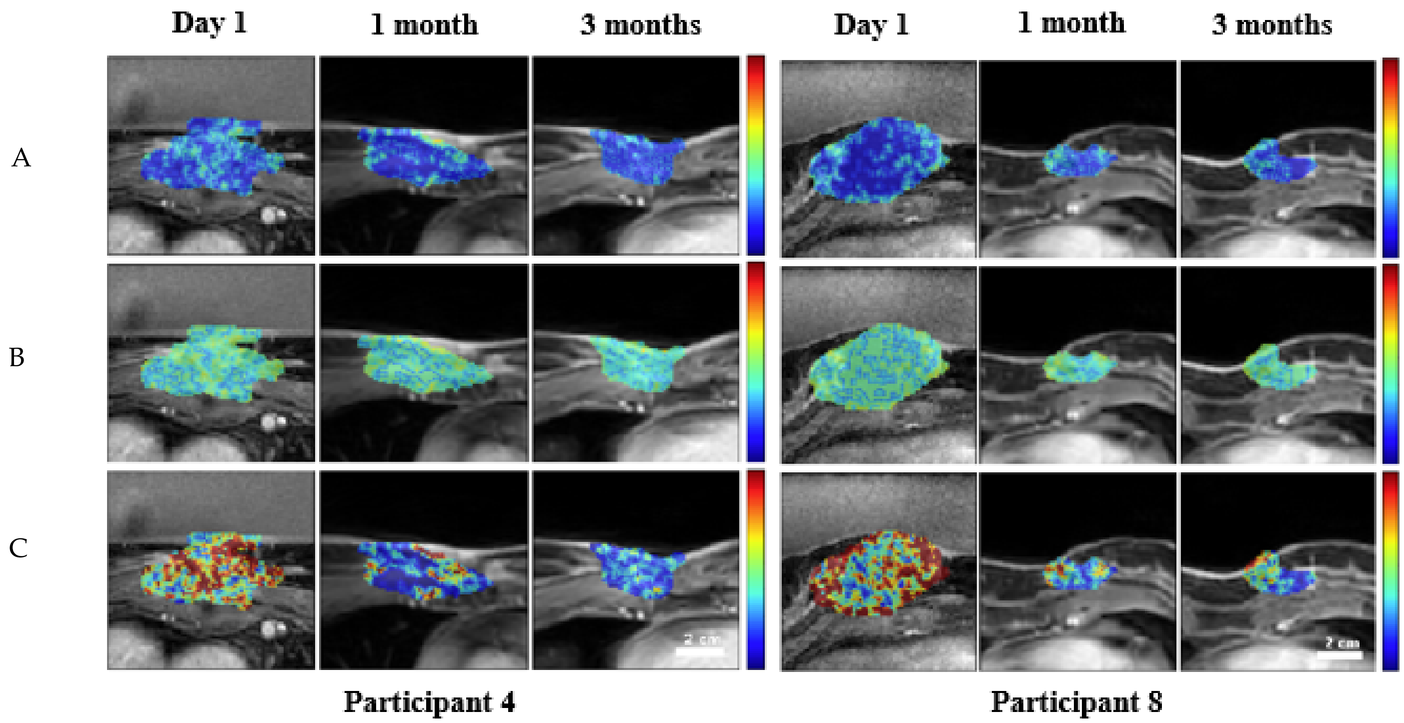

Figure S3. Two dimensional feature texture maps (1.0 mm spacing) demonstrating 3 significant features for 2 patients on 1st day of treatment (immediately prior to treatment) and over 1 month and 3 months follow-up in the following order (starting from 1st row): HH wavelet glszm Zone Percentage (scale range = 0.0- 1.4)A.U, HH wavelet glrlm Run length non-uniformity (scale range= 0.0 – 1.4)A.U and HH wavelet 1st order Interquartile Range (scale range = 0.0 - 20) A.U. The individual features colour bar scale is consistent for both patients. The scale bar on the right bottom corner represents 2 cm.

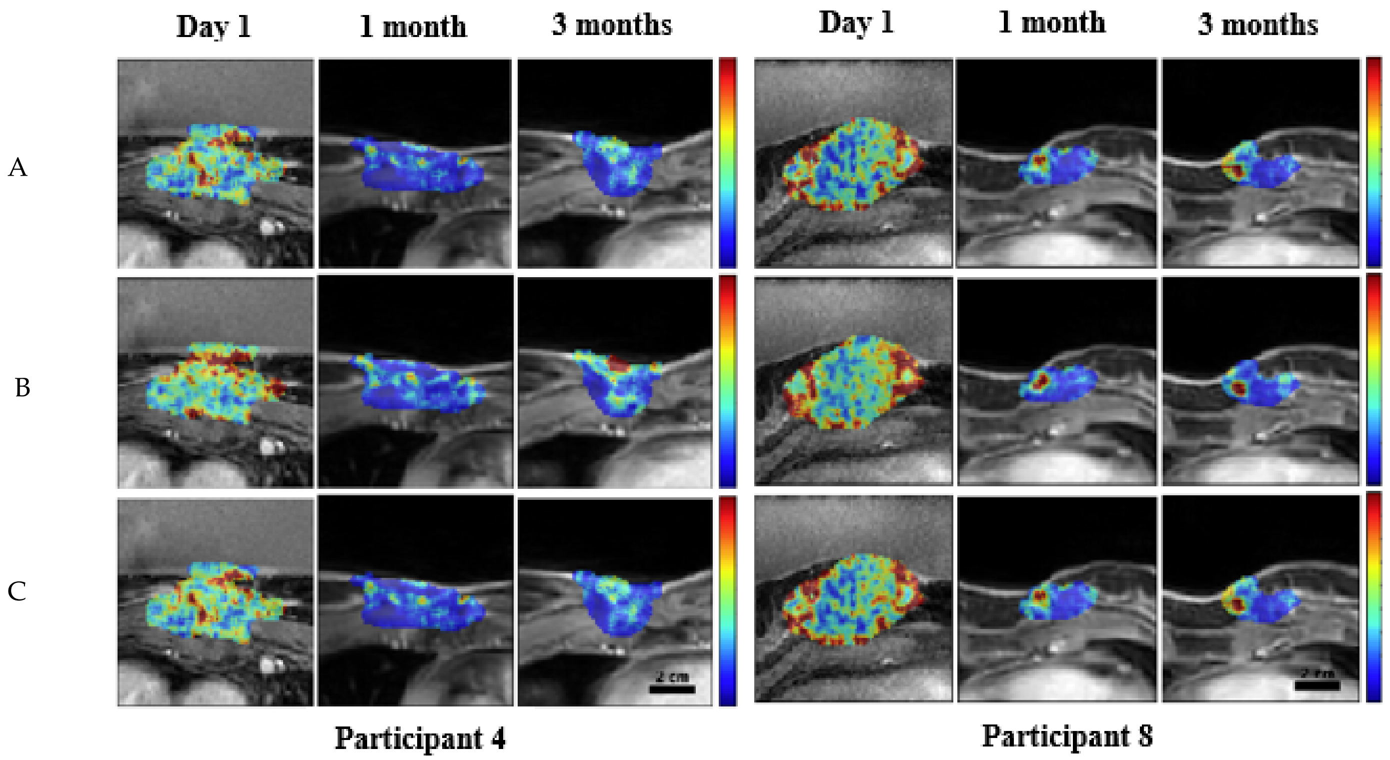

Figure S4. Three dimensional feature texture maps (1.0 mm spacing) demonstrating 3 significant features for the same set of 2 patients on 1st day of treatment (prior to treatment) and over 1 month and 3 months follow-up in the following order (starting from 1st row): A. HHL wavelet 1st order

Interquartile Range (scale range= 0.0 - 45)A.U, B. HHH wavelet 1st order Interquartile Range (scale range= 0.0 - 5.0)A.U and C. HHL wavelet 1st order Robust mean absolute deviation (scale range = 0.0 -18.0) A.U. The scale bar on the right bottom corner represents 2 cm.

Table S2. The 2D features with raw p- value, corrected p-value and effect sizes. (grid separation of 0.5 mm).

Feature	P-Value	P-Value-Adjusted	Cohens d 1month day1	Cohens d 3month day1
original_shape_MinorAxisLength	0.0001033	0.034182716	1.099218763	1.692640396
original_firstorder_Mean	5.868E-05	0.01942291	1.657921152	1.278257715
original_firstorder_Median	3.608E-05	0.01194379	1.896308616	1.329539938
original_firstorder_Minimum	1.191E-08	3.94211E-06	2.897631641	2.567296563
original_firstorder_RootMeanSquared	3.879E-05	0.012840851	1.718696889	1.286942389
original_glrlm_LongRunHighGrayLevelEmphasis	2.476E-06	0.000819534	2.20687113	1.766791586
wavelet-LH_firstorder_90Percentile	3.639E-06	0.001204357	1.861975924	1.842317557
wavelet-LH_firstorder_Entropy	5.635E-05	0.018650436	1.366129196	1.668493134
wavelet-LH_firstorder_InterquartileRange	3.578E-06	0.00118429	1.807809308	1.742211392
wavelet-LH_firstorder_MeanAbsoluteDeviation	5.744E-06	0.001901302	1.680706321	1.735527243
wavelet-LH_firstorder_Minimum	3.51E-05	0.011617135	1.472009773	1.443246282
wavelet-LH_firstorder_Range	6.196E-05	0.020508982	1.525288771	1.324979934
wavelet-LH_firstorder_RobustMeanAbsoluteDeviation	3.177E-06	0.001051494	1.794327592	1.795423671
wavelet-LH_firstorder_RootMeanSquared	1.531E-05	0.005067007	1.536017273	1.595278438
wavelet-LH_firstorder_Variance	1.37E-05	0.004534014	1.684799779	1.437445748
wavelet-LH_glcm_JointAverage	2.556E-05	0.008459024	1.476009496	1.537811048
wavelet-LH_glcm_ClusterTendency	7.27E-06	0.002406295	1.668164755	1.546436154
wavelet-LH_glcm_Contrast	4.936E-06	0.001633937	1.80528382	1.604310102
wavelet-LH_glcm_DifferenceAverage	1.834E-06	0.000607012	1.798980263	1.940490282
wavelet-LH_glcm_DifferenceEntropy	1.945E-05	0.006436389	1.473860595	1.857019272
wavelet-LH_glcm_DifferenceVariance	1.811E-05	0.005995597	1.633441297	1.465277619
wavelet-LH_glcm_JointEntropy	1.208E-05	0.0039994	1.538351492	1.893026834
wavelet-LH_glcm_Imc1	4.726E-11	1.56446E-08	4.996615462	3.641635976
wavelet-LH_glcm_Imc2	4.78E-06	0.001582116	1.758234707	1.943764642
wavelet-LH_glcm_Idm	1.483E-05	0.00490958	1.599593748	1.745869795
wavelet-LH_glcm_Id	1.145E-05	0.003791067	1.617989848	1.791045925
wavelet-LH_glcm_InverseVariance	6.468E-06	0.002140812	1.63275305	1.792041499
wavelet-LH_glcm_MaximumProbability	0.0001317	0.043598114	1.411519655	1.49382581
wavelet-LH_glcm_SumEntropy	2.626E-05	0.008693381	1.428751643	1.733948487
wavelet-LH_glcm_SumSquares	5.61E-06	0.001856783	1.738576107	1.5810261
wavelet-LH_glrlm_GrayLevelVariance	6.122E-05	0.02026245	1.475857948	1.282925378
wavelet-LH_glrlm_HighGrayLevelRunEmphasis	0.0001129	0.03737562	1.286584517	1.273668273
wavelet-LH_glrlm_RunLengthNonUniformityNormalized	5.35E-07	0.000177094	2.034871445	2.091526398
wavelet-LH_glrlm_RunPercentage	9.198E-07	0.000304466	1.984766789	2.014337298
wavelet-LH_glrlm_RunVariance	0.0001075	0.035581952	1.45463393	1.433777089
wavelet-LH_glrlm_ShortRunEmphasis	6.827E-06	0.002259615	1.709682482	1.841913546
wavelet-LH_glrlm_ShortRunHighGrayLevelEmphasis	3.18E-05	0.010524188	1.422012402	1.407187668
wavelet-LH_glszm_HighGrayLevelZoneEmphasis	9.091E-05	0.030089592	1.309412691	1.301923507
wavelet-LH_glszm_SizeZoneNonUniformityNormalized	5.658E-07	0.000187265	1.914313518	2.197993318
wavelet-LH_glszm_SmallAreaEmphasis	1.243E-05	0.004113721	1.53727227	1.830288427
wavelet-LH_glszm_SmallAreaHighGrayLevelEmphasis	1.802E-05	0.00596309	1.476072887	1.480406745
wavelet-LH_glszm_ZoneEntropy	1.6E-06	0.000529718	2.257473642	1.436802521
wavelet-LH_glszm_ZonePercentage	1.083E-09	3.58455E-07	3.062530012	2.891504547
wavelet-LH_gldm_DependenceNonUniformityNormalized	5.022E-06	0.001662291	1.718301708	1.624189491
wavelet-LH_gldm_DependenceVariance	1.556E-05	0.005151589	1.947572009	1.414583235
wavelet-LH_gldm_GrayLevelVariance	1.38E-05	0.004569297	1.684388079	1.437050291
wavelet-LH_gldm_HighGrayLevelEmphasis	0.0001214	0.040187297	1.279426005	1.26412824
wavelet-LH_gldm_SmallDependenceEmphasis	1.924E-07	6.36951E-05	2.128420147	2.003278203

wavelet-LH_gldm_SmallDependenceHighGrayLevelEmphasis	8.803E-07	0.000291384	1.940656339	1.768797201
wavelet-LH_ngtdm_Complexity	2.08E-05	0.006884711	1.437531248	1.495245258
wavelet-HL_firstorder_10Percentile	9.455E-06	0.003129594	1.728288767	1.446434548
wavelet-HL_firstorder_90Percentile	1.56E-06	0.000516496	1.889764851	1.713063387
wavelet-HL_firstorder_Entropy	6.214E-06	0.002056743	1.767394632	1.59401134
wavelet-HL_firstorder_InterquartileRange	2.365E-07	7.82718E-05	2.097987762	1.950995822
wavelet-HL_firstorder_Maximum	6.229E-05	0.02061891	1.398823778	1.506724577
wavelet-HL_firstorder_MeanAbsoluteDeviation	1.833E-06	0.000606647	1.865509506	1.69980995
wavelet-HL_firstorder_RobustMeanAbsoluteDeviation	1.983E-07	6.56288E-05	2.118962987	1.997001676
wavelet-HL_firstorder_RootMeanSquared	1.682E-05	0.005568099	1.607745051	1.450329013
wavelet-HL_firstorder_Skewness	1.242E-05	0.00410937	1.964918259	1.706834793
wavelet-HL_firstorder_Uniformity	5.704E-06	0.001887888	1.778008425	1.632576337
wavelet-HL_firstorder_Variance	2.424E-05	0.008024876	1.530542904	1.370454945
wavelet-HL_glcm_ClusterShade	8.983E-06	0.002973536	1.536538059	1.598784262
wavelet-HL_glcm_ClusterTendency	2.453E-05	0.008120912	1.531274033	1.363241642
wavelet-HL_glcm_Contrast	1.675E-05	0.005543712	1.557145003	1.429798194
wavelet-HL_glcm_DifferenceAverage	1.041E-06	0.000344429	1.945427428	1.78266029
wavelet-HL_glcm_DifferenceEntropy	3.328E-06	0.00110168	1.869634528	1.676341597
wavelet-HL_glcm_DifferenceVariance	0.0001155	0.038215021	1.334737486	1.227224909
wavelet-HL_glcm_JointEnergy	6.523E-06	0.002159135	1.769139041	1.639798883
wavelet-HL_glcm_JointEntropy	1.376E-06	0.000455505	1.96206064	1.775586589
wavelet-HL_glcm_Imc1	4.766E-09	1.5774E-06	2.820512044	3.383058339
wavelet-HL_glcm_Imc2	1.55E-06	0.000513047	1.867884804	2.17671348
wavelet-HL_glcm_Idm	1.136E-07	3.76128E-05	2.320523416	2.136268224
wavelet-HL_glcm_Id	1.258E-07	4.16308E-05	2.301622769	2.11663946
wavelet-HL_glcm_InverseVariance	4.469E-07	0.000147932	2.07720246	1.913584652
wavelet-HL_glcm_MaximumProbability	7.483E-07	0.000247676	2.084577329	1.984421145
wavelet-HL_glcm_SumEntropy	2.644E-06	0.000875214	1.887562356	1.67907564
wavelet-HL_glcm_SumSquares	2.018E-05	0.006679538	1.544570167	1.39666127
wavelet-HL_glrlm_GrayLevelNonUniformityNormalized	3.738E-05	0.012373936	1.5833248	1.429564929
wavelet-HL_glrlm_GrayLevelVariance	0.0001116	0.036941931	1.347649443	1.22902415
wavelet-HL_glrlm_LongRunEmphasis	1.696E-06	0.000561326	2.151387705	1.717720439
wavelet-HL_glrlm_RunLengthNonUniformityNormalized	8.91E-10	2.94917E-07	3.359902448	2.887066858
wavelet-HL_glrlm_RunPercentage	1.035E-09	3.42619E-07	3.469084033	2.771346986
wavelet-HL_glrlm_RunVariance	1.868E-06	0.000618334	2.167112	1.63204401
wavelet-HL_glrlm_ShortRunEmphasis	1.114E-08	3.68729E-06	2.982543349	2.488698318
wavelet-HL_glszm_SizeZoneNonUniformityNormalized	4.419E-08	1.46272E-05	2.582808373	2.341554587
wavelet-HL_glszm_SmallAreaEmphasis	2.795E-07	9.25181E-05	2.401522428	2.128445665
wavelet-HL_glszm_ZoneEntropy	1.985E-09	6.5718E-07	3.357794499	2.508843061
wavelet-HL_glszm_ZonePercentage	2.337E-11	7.73675E-09	3.946426906	3.556652131
wavelet-HL_gldm_DependenceNonUniformityNormalized	2.135E-09	7.06749E-07	2.863856419	2.817057756
wavelet-HL_gldm_DependenceVariance	1.093E-08	3.61716E-06	3.27244578	2.411204354
wavelet-HL_gldm_GrayLevelVariance	2.514E-05	0.008322199	1.525675163	1.367118924
wavelet-HL_gldm_LargeDependenceEmphasis	7.081E-07	0.000234376	2.1346965	1.837895577
wavelet-HL_gldm_SmallDependenceEmphasis	6.979E-08	2.31017E-05	2.237825146	2.224283858
wavelet-HH_firstorder_10Percentile	3.347E-13	1.1079E-10	4.882663349	4.542799261
wavelet-HH_firstorder_90Percentile	1.975E-13	6.53631E-11	5.108213312	4.585116846
wavelet-HH_firstorder_Entropy	2.867E-08	9.48942E-06	2.402714978	2.250896856
wavelet-HH_firstorder_InterquartileRange	3.578E-14	1.18446E-11	5.602388418	5.161856118
wavelet-HH_firstorder_MeanAbsoluteDeviation	1.962E-12	6.4944E-10	4.448432522	4.005762674
wavelet-HH_firstorder_Median	1.542E-07	5.10527E-05	2.280473889	1.992464359
wavelet-HH_firstorder_Minimum	1.937E-06	0.000641231	1.857049184	1.880085783
wavelet-HH_firstorder_Range	3.01E-05	0.009961778	1.483788845	1.52661101
wavelet-HH_firstorder_RobustMeanAbsoluteDeviation	2.984E-14	9.87682E-12	5.638483147	5.206281844
wavelet-HH_firstorder_RootMeanSquared	3.216E-10	1.06438E-07	3.364433728	2.89405483
wavelet-HH_firstorder_Uniformity	5.32E-08	1.76086E-05	2.258811938	2.190935723

wavelet-HH_firstorder_Variance	6.075E-08	2.01074E-05	2.266754091	2.130501162
wavelet-HH_glcm_JointAverage	1.417E-06	0.000468916	1.953229689	1.946963168
wavelet-HH_glcm_ClusterShade	2.551E-08	8.44255E-06	2.848945056	2.294195311
wavelet-HH_glcm_ClusterTendency	8.728E-06	0.002888941	1.590487131	1.503373407
wavelet-HH_glcm_Contrast	8.338E-06	0.00275982	1.594716479	1.509517342
wavelet-HH_glcm_DifferenceAverage	4.525E-07	0.000149788	1.965895492	1.873155368
wavelet-HH_glcm_DifferenceEntropy	2.278E-08	7.54131E-06	2.476610798	2.253842949
wavelet-HH_glcm_DifferenceVariance	1.84E-05	0.006090694	1.511070016	1.409723029
wavelet-HH_glcm_JointEnergy	1.157E-08	3.82917E-06	2.514238086	2.409406326
wavelet-HH_glcm_JointEntropy	1.916E-08	6.34316E-06	2.468997463	2.302783717
wavelet-HH_glcm_Idm	6.725E-08	2.22594E-05	2.231317639	2.14135664
wavelet-HH_glcm_Id	5.526E-08	1.82924E-05	2.259827576	2.170767787
wavelet-HH_glcm_MaximumProbability	2.618E-07	8.6653E-05	1.988832861	2.022978439
wavelet-HH_glcm_SumEntropy	2.431E-08	8.04719E-06	2.450459193	2.252672141
wavelet-HH_glcm_SumSquares	8.527E-06	0.002822283	1.592626713	1.506500813
wavelet-HH_glrlm_GrayLevelNonUniformityNormalized	3.464E-09	1.14645E-06	2.786600463	2.580957679
wavelet-HH_glrlm_GrayLevelVariance	2.968E-06	0.000982365	1.75627224	1.607135668
wavelet-HH_glrlm_HighGrayLevelRunEmphasis	9.546E-06	0.00315987	1.554992464	1.597179837
wavelet-HH_glrlm_LongRunEmphasis	1.495E-09	4.94863E-07	3.031342655	2.862966897
wavelet-HH_glrlm_RunLengthNonUniformityNormalized	3.231E-09	1.0693E-06	2.861213178	2.654967079
wavelet-HH_glrlm_RunPercentage	3.573E-10	1.18282E-07	3.22311631	3.063643143
wavelet-HH_glrlm_RunVariance	8.701E-09	2.88006E-06	2.715102366	2.5091282
wavelet-HH_glrlm_ShortRunEmphasis	7.813E-10	2.58617E-07	3.13308601	2.845762211
wavelet-HH_glrlm_ShortRunHighGrayLevelEmphasis	6.837E-06	0.002263052	1.579679934	1.595536343
wavelet-HH_glszm_GrayLevelVariance	5.133E-08	1.69907E-05	2.959486097	1.920148415
wavelet-HH_glszm_HighGrayLevelZoneEmphasis	1.378E-05	0.004560616	1.519445235	1.539976151
wavelet-HH_glszm_SizeZoneNonUniformityNormalized	6.064E-06	0.002007102	1.862271448	1.616185209
wavelet-HH_glszm_SmallAreaEmphasis	3.461E-05	0.011456986	1.583087126	1.465903584
wavelet-HH_glszm_SmallAreaHighGrayLevelEmphasis	5.391E-06	0.001784382	1.635785925	1.635188387
wavelet-HH_glszm_ZoneEntropy	2.39E-05	0.0079105	1.739299028	1.544392173
wavelet-HH_glszm_ZonePercentage	2.532E-07	8.38015E-05	2.053196264	1.945672236
wavelet-HH_gldm_DependenceVariance	1.684E-08	5.57285E-06	2.69503413	2.312894451
wavelet-HH_gldm_GrayLevelVariance	1.128E-05	0.00373452	1.555493583	1.477475037
wavelet-HH_gldm_HighGrayLevelEmphasis	9.186E-06	0.003040672	1.558953077	1.603274853
wavelet-HH_gldm_LargeDependenceEmphasis	3.28E-06	0.001085546	1.900773954	1.530231363
wavelet-HH_gldm_LargeDependenceHighGrayLevelEmphasis	3.043E-05	0.01007342	1.43386314	1.496557245
wavelet-HH_gldm_SmallDependenceEmphasis	1.161E-12	3.84409E-10	4.295818619	4.470512157
wavelet-HH_gldm_SmallDependenceHighGrayLevelEmphasis	3.944E-07	0.000130534	1.94896353	1.917756731
wavelet-HH_ngtdm_Complexity	7.289E-05	0.02412737	1.295348694	1.354916909
wavelet-LL_firstorder_Mean	5.87E-05	0.019429371	1.657858643	1.278014278
wavelet-LL_firstorder_Median	3.534E-05	0.011698046	1.897346609	1.33058093
wavelet-LL_firstorder_Minimum	1.393E-08	4.60925E-06	2.379549809	2.924606656
wavelet-LL_firstorder_RootMeanSquared	3.778E-05	0.012506244	1.723014092	1.289396067

Table S3. The 3D features with raw p- value, corrected p-value and effect sizes. (grid separation of 0.5 mm).

Feature	P-Value	P-Value-Adjusted	Cohens d 1month day1	Cohens d 3month day1
original_shape_MinorAxisLength	9.51E-05	0.049542	1.111148	1.688115
original_firstorder_Mean	6.45E-05	0.033617	1.650237	1.263223

original_firstorder_Median	3.57E-05	0.01861	1.897692	1.327355
original_firstorder_Minimum	8.12E-08	4.23E-05	2.713788	2.388785
original_firstorder_RootMeanSquared	4.04E-05	0.021058	1.716229	1.279028
wavelet-LLH_firstorder_90Percentile	3.53E-05	0.018372	2.355966	1.423028
wavelet-LLH_firstorder_Entropy	7.91E-05	0.041222	2.323161	1.440575
wavelet-LLH_firstorder_InterquartileRange	3.53E-05	0.018376	1.974128	1.387635
wavelet-LLH_firstorder_RobustMeanAbsoluteDeviation	3.91E-05	0.020397	2.042725	1.37304
wavelet-LLH_glcm_DifferenceEntropy	9.39E-05	0.048924	2.283223	1.497577
wavelet-LLH_glcm_JointEntropy	2.23E-05	0.011631	2.45399	1.585916
wavelet-LLH_glcm_Idm	4.28E-05	0.022293	1.815339	1.699991
wavelet-LLH_glcm_Id	3.24E-05	0.016887	2.045375	1.687973
wavelet-LLH_glcm_InverseVariance	8.09E-05	0.042131	1.981678	1.522547
wavelet-LLH_glrlm_RunLengthNonUniformityNormalized	3.65E-06	0.0019	2.011155	2.069093
wavelet-LLH_glrlm_RunPercentage	1.27E-05	0.006591	1.607855	2.028645
wavelet-LLH_glszm_SizeZoneNonUniformityNormalized	4.62E-05	0.024096	2.145988	1.621689
wavelet-LLH_glszm_SmallAreaEmphasis	8.01E-05	0.041707	1.719849	1.52879
wavelet-LLH_glszm_ZonePercentage	1.5E-05	0.007824	2.258001	1.531329
wavelet-LHL_firstorder_Minimum	7.19E-05	0.037474	1.19103	2.228823
wavelet-LHL_firstorder_Range	5.08E-05	0.026488	1.382554	1.77679
wavelet-LHL_glcm_JointAverage	6.1E-05	0.031774	1.217213	2.239724
wavelet-LHL_glrlm_HighGrayLevelRunEmphasis	7.34E-05	0.038229	1.154023	1.960778
wavelet-LHL_glrlm_ShortRunHighGrayLevelEmphasis	5.83E-05	0.030374	1.180909	1.984401
wavelet-LHL_glszm_HighGrayLevelZoneEmphasis	7.11E-05	0.037062	1.156992	1.966973
wavelet-LHL_glszm_SmallAreaHighGrayLevelEmphasis	3.96E-05	0.020626	1.226594	1.985649
wavelet-LHL_glszm_ZonePercentage	4.57E-05	0.023791	1.582901	1.486362
wavelet-LHL_gldm_HighGrayLevelEmphasis	7.35E-05	0.038291	1.153828	1.96072
wavelet-LHL_gldm_SmallDependenceHighGrayLevelEmphasis	7.56E-05	0.039406	1.659058	1.381665
wavelet-LHH_firstorder_10Percentile	1.54E-07	8.05E-05	2.535345	2.037116
wavelet-LHH_firstorder_90Percentile	4.15E-07	0.000216	2.462009	2.000327
wavelet-LHH_firstorder_Entropy	1.35E-06	0.000703	1.917878	2.110212
wavelet-LHH_firstorder_InterquartileRange	3.4E-08	1.77E-05	2.797265	2.214446
wavelet-LHH_firstorder_MeanAbsoluteDeviation	2.86E-07	0.000149	2.482179	1.962892
wavelet-LHH_firstorder_Median	3.31E-05	0.017251	1.421303	1.404008

wavelet-LHH_firstorder_RobustMeanAbsoluteDeviation	4.07E-08	2.12E-05	2.773666	2.206968
wavelet-LHH_firstorder_RootMeanSquared	2.78E-06	0.001446	2.09571	1.68577
wavelet-LHH_firstorder_Uniformity	1.11E-05	0.005775	1.555554	2.036731
wavelet-LHH_firstorder_Variance	4.92E-05	0.025653	1.683622	1.252837
wavelet-LHH_glcm_ClusterTendency	1.32E-05	0.00688	1.897198	1.431287
wavelet-LHH_glcm_Contrast	1.31E-05	0.006816	1.898876	1.431942
wavelet-LHH_glcm_DifferenceAverage	7.58E-07	0.000395	2.17962	1.948564
wavelet-LHH_glcm_DifferenceEntropy	3.04E-06	0.001586	1.762424	2.051481
wavelet-LHH_glcm_DifferenceVariance	5.18E-05	0.026997	1.656727	1.304891
wavelet-LHH_glcm_JointEntropy	1.56E-06	0.000811	1.84424	2.156875
wavelet-LHH_glcm_Idm	1.97E-06	0.001025	1.749654	2.169896
wavelet-LHH_glcm_Id	1.64E-06	0.000857	1.786953	2.179527
wavelet-LHH_glcm_InverseVariance	5.17E-07	0.000269	2.045188	2.146959
wavelet-LHH_glcm_MaximumProbability	1.56E-05	0.008149	1.485508	1.908518
wavelet-LHH_glcm_SumEntropy	3.44E-06	0.001793	1.732691	2.06077
wavelet-LHH_glcm_SumSquares	1.31E-05	0.006848	1.898042	1.431615
wavelet-LHH_glrlm_GrayLevelNonUniformityNormalized	9.79E-06	0.005099	1.576439	2.277779
wavelet-LHH_glrlm_GrayLevelVariance	8.26E-05	0.043017	1.618386	1.208283
wavelet-LHH_glrlm_LongRunEmphasis	2.73E-05	0.014228	1.510477	1.950662
wavelet-LHH_glrlm_RunLengthNonUniformityNormalized	1.32E-06	0.000688	1.810416	2.119895
wavelet-LHH_glrlm_RunPercentage	1.61E-06	0.000841	1.79619	2.104878
wavelet-LHH_glrlm_RunVariance	3.04E-05	0.015839	1.592358	1.643665
wavelet-LHH_glrlm_ShortRunEmphasis	3.63E-06	0.001892	1.679013	2.196861
wavelet-LHH_glszm_ZonePercentage	4.99E-07	0.00026	2.198978	1.908637
wavelet-LHH_gldm_DependenceNonUniformityNormalized	1.19E-06	0.000619	2.026621	1.763101
wavelet-LHH_gldm_GrayLevelVariance	5.14E-05	0.026789	1.67079	1.249231
wavelet-HLL_glszm_ZonePercentage	2.58E-06	0.001347	1.804696	1.753155
wavelet-HLH_firstorder_10Percentile	2.01E-06	0.001049	2.059462	1.562018
wavelet-HLH_firstorder_90Percentile	2.25E-06	0.00117	2.057811	1.556295
wavelet-HLH_firstorder_Entropy	6.64E-06	0.003459	1.898361	1.515474
wavelet-HLH_firstorder_InterquartileRange	5.74E-07	0.000299	2.103041	1.743598
wavelet-HLH_firstorder_MeanAbsoluteDeviation	2.71E-06	0.001414	2.000132	1.547161

wavelet-HLH_firstorder_Median	1.76E-06	0.000918	1.745627	1.791983
wavelet-HLH_firstorder_Minimum	4.4E-05	0.022911	1.539698	1.497577
wavelet-HLH_firstorder_RobustMeanAbsoluteDeviation	5.96E-07	0.00031	2.119772	1.732242
wavelet-HLH_firstorder_RootMeanSquared	1.52E-05	0.00794	1.815075	1.396652
wavelet-HLH_firstorder_Uniformity	5.5E-06	0.002867	1.866198	1.582336
wavelet-HLH_firstorder_Variance	4.09E-05	0.021328	1.590237	1.266089
wavelet-HLH_glcm_JointAverage	5.63E-05	0.029349	1.478225	1.498077
wavelet-HLH_glcm_ClusterShade	1.43E-06	0.000746	1.824406	1.973616
wavelet-HLH_glcm_ClusterTendency	3.11E-05	0.016222	1.629836	1.289805
wavelet-HLH_glcm_Contrast	3.1E-05	0.016162	1.63086	1.289397
wavelet-HLH_glcm_DifferenceAverage	5E-06	0.002603	1.916554	1.489849
wavelet-HLH_glcm_DifferenceEntropy	8.28E-06	0.004315	1.898543	1.500407
wavelet-HLH_glcm_DifferenceVariance	9.04E-05	0.047102	1.521096	1.189641
wavelet-HLH_glcm_JointEnergy	1.73E-05	0.009012	1.656092	1.550935
wavelet-HLH_glcm_JointEntropy	4.82E-06	0.002509	1.925916	1.557504
wavelet-HLH_glcm_Idm	1.94E-06	0.001008	2.019753	1.650278
wavelet-HLH_glcm_Id	2.03E-06	0.001058	2.014799	1.639897
wavelet-HLH_glcm_InverseVariance	2.84E-06	0.001478	2.018104	1.575072
wavelet-HLH_glcm_MaximumProbability	3.32E-06	0.001731	1.89846	1.61411
wavelet-HLH_glcm_SumEntropy	6.8E-06	0.003541	1.903652	1.526184
wavelet-HLH_glcm_SumSquares	3.11E-05	0.016192	1.630349	1.289601
wavelet-HLH_glrlm_GrayLevelNonUniformityNormalized	1.1E-05	0.005742	1.799306	1.558654
wavelet-HLH_glrlm_GrayLevelVariance	8.71E-05	0.045404	1.502209	1.208343
wavelet-HLH_glrlm_LongRunEmphasis	2.82E-06	0.001467	1.825085	1.900285
wavelet-HLH_glrlm_RunLengthNonUniformityNormalized	2.09E-07	0.000109	2.327999	1.963974
wavelet-HLH_glrlm_RunPercentage	1.65E-07	8.6E-05	2.375639	2.010359
wavelet-HLH_glrlm_RunVariance	2.53E-06	0.001318	1.899837	1.825879
wavelet-HLH_glrlm_ShortRunEmphasis	3.23E-07	0.000168	2.228231	2.06128
wavelet-HLH_glszm_ZonePercentage	1.41E-06	0.000737	1.984244	1.659975
wavelet-HLH_gldm_DependenceNonUniformityNormalized	3.84E-06	0.002002	2.098055	2.677462
wavelet-HLH_gldm_DependenceVariance	1.27E-05	0.006604	2.207438	1.277866
wavelet-HLH_gldm_GrayLevelVariance	4.41E-05	0.022959	1.580053	1.259692
wavelet-HLH_gldm_SmallDependenceEmphasis	1.18E-05	0.006124	1.791113	1.370423
wavelet-HLH_gldm_SmallDependenceHighGrayLevelEmphasis	2.84E-05	0.014786	1.582759	1.327606
wavelet-HHL_firstorder_10Percentile	7.48E-09	3.9E-06	2.583124	2.51206

wavelet-HHL_firstorder_90Percentile	1.09E-08	5.66E-06	2.527394	2.467649
wavelet-HHL_firstorder_Entropy	2.7E-10	1.4E-07	3.597267	3.080607
wavelet-HHL_firstorder_InterquartileRange	2.41E-09	1.26E-06	2.784676	2.70976
wavelet-HHL_firstorder_MeanAbsoluteDeviation	1.02E-08	5.3E-06	2.545747	2.437899
wavelet-HHL_firstorder_RobustMeanAbsoluteDeviation	2.48E-09	1.29E-06	2.763805	2.718193
wavelet-HHL_firstorder_RootMeanSquared	6.36E-08	3.31E-05	2.320004	2.11045
wavelet-HHL_firstorder_Variance	1.09E-06	0.000568	1.858106	1.745326
wavelet-HHL_glcm_ClusterShade	1.18E-05	0.006162	1.485507	1.820031
wavelet-HHL_glcm_ClusterTendency	3.16E-07	0.000165	2.008763	1.921029
wavelet-HHL_glcm_Contrast	3.11E-07	0.000162	2.009911	1.924159
wavelet-HHL_glcm_DifferenceAverage	8.18E-09	4.26E-06	2.579592	2.479391
wavelet-HHL_glcm_DifferenceEntropy	7.15E-10	3.72E-07	3.313423	2.9173
wavelet-HHL_glcm_DifferenceVariance	1.27E-06	0.00066	1.871955	1.701611
wavelet-HHL_glcm_JointEntropy	8.69E-11	4.53E-08	3.91425	3.213504
wavelet-HHL_glcm_Idm	6.32E-09	3.29E-06	3.012743	2.384538
wavelet-HHL_glcm_Id	3.68E-10	1.91E-07	3.661867	2.884113
wavelet-HHL_glcm_InverseVariance	2.14E-08	1.12E-05	2.697347	2.339272
wavelet-HHL_glcm_SumEntropy	3.07E-10	1.6E-07	3.530787	3.060041
wavelet-HHL_glcm_SumSquares	3.13E-07	0.000163	2.009338	1.922597
wavelet-HHL_glrlm_GrayLevelNonUniformityNormalized	3.44E-05	0.017907	1.477425	1.319723
wavelet-HHL_glrlm_GrayLevelVariance	1.29E-06	0.000671	1.8439	1.719581
wavelet-HHL_glrlm_RunEntropy	1.68E-06	0.000875	1.833656	1.840186
wavelet-HHL_glrlm_RunLengthNonUniformityNormalized	9.11E-08	4.75E-05	2.357543	2.038496
wavelet-HHL_glrlm_RunPercentage	1.19E-05	0.006195	1.576909	1.472104
wavelet-HHL_glrlm_ShortRunEmphasis	9.1E-05	0.047424	1.316257	1.255298
wavelet-HHL_glszm_GrayLevelVariance	7.14E-06	0.003722	1.71699	1.458873
wavelet-HHL_glszm_SizeZoneNonUniformityNormalized	1.71E-07	8.88E-05	2.001007	2.667046
wavelet-HHL_glszm_SmallAreaEmphasis	1.9E-06	0.000989	1.734396	3.289213
wavelet-HHL_glszm_ZonePercentage	1.55E-08	8.1E-06	2.545224	2.398634
wavelet-HHL_gldm_DependenceNonUniformityNormalized	1.58E-07	8.23E-05	2.226335	2.230853
wavelet-HHL_gldm_GrayLevelVariance	1.1E-06	0.000575	1.856448	1.744172
wavelet-HHL_gldm_SmallDependenceEmphasis	2.29E-06	0.001195	1.841186	1.63268
wavelet-HHL_ngtdm_Complexity	6.79E-05	0.035373	1.353857	1.273041
wavelet-HHL_ngtdm_Contrast	4.27E-05	0.022233	1.338694	1.523283

wavelet-HHH_firstorder_10Percentile	6.17E-08	3.21E-05	2.421224	2.099203
wavelet-HHH_firstorder_90Percentile	9.67E-08	5.04E-05	2.388434	2.013151
wavelet-HHH_firstorder_Entropy	4.27E-07	0.000223	2.234597	1.822188
wavelet-HHH_firstorder_InterquartileRange	1.17E-08	6.1E-06	2.569911	2.368215
wavelet-HHH_firstorder_MeanAbsoluteDeviation	1.72E-07	8.94E-05	2.33195	1.915616
wavelet-HHH_firstorder_Median	5.33E-06	0.002779	1.663571	1.664696
wavelet-HHH_firstorder_RobustMeanAbsoluteDeviation	1.63E-08	8.5E-06	2.54519	2.302624
wavelet-HHH_firstorder_RootMeanSquared	3.24E-06	0.001688	2.034813	1.523879
wavelet-HHH_firstorder_Uniformity	1.16E-07	6.02E-05	2.348464	2.038653
wavelet-HHH_firstorder_Variance	3.23E-05	0.016825	1.59134	1.27809
wavelet-HHH_glcm_ClusterTendency	1.26E-05	0.006577	1.723333	1.384322
wavelet-HHH_glcm_Contrast	1.27E-05	0.006608	1.722703	1.383802
wavelet-HHH_glcm_DifferenceAverage	4.59E-07	0.000239	2.1656	1.804675
wavelet-HHH_glcm_DifferenceEntropy	6.73E-07	0.000351	2.199842	1.770538
wavelet-HHH_glcm_DifferenceVariance	5.66E-05	0.029473	1.637385	1.192646
wavelet-HHH_glcm_JointEnergy	1.35E-07	7.04E-05	2.279955	2.088038
wavelet-HHH_glcm_JointEntropy	2.41E-07	0.000125	2.281898	1.926698
wavelet-HHH_glcm_Idm	8.05E-08	4.19E-05	2.363438	2.097255
wavelet-HHH_glcm_Id	8.01E-08	4.17E-05	2.366733	2.098377
wavelet-HHH_glcm_InverseVariance	7.6E-05	0.039597	1.407227	1.230874
wavelet-HHH_glcm_MaximumProbability	3.04E-08	1.58E-05	2.514649	2.276626
wavelet-HHH_glcm_SumEntropy	4.03E-07	0.00021	2.244778	1.849408
wavelet-HHH_glcm_SumSquares	1.27E-05	0.006593	1.723018	1.384062
wavelet-HHH_glrlm_GrayLevelNonUniformityNormalized	5.35E-07	0.000279	2.157214	1.845964
wavelet-HHH_glrlm_GrayLevelVariance	7.12E-05	0.037087	1.549863	1.176251
wavelet-HHH_glrlm_LongRunEmphasis	1.67E-08	8.7E-06	2.547833	2.622472
wavelet-HHH_glrlm_RunLengthNonUniformityNormalized	2.29E-08	1.19E-05	2.487293	2.379806
wavelet-HHH_glrlm_RunPercentage	1.61E-08	8.4E-06	2.522896	2.465432
wavelet-HHH_glrlm_RunVariance	4.75E-08	2.47E-05	2.316448	2.408028
wavelet-HHH_glrlm_ShortRunEmphasis	2.42E-08	1.26E-05	2.503219	2.43825
wavelet-HHH_glszm_ZonePercentage	3.12E-06	0.001627	1.83438	1.591377
wavelet-HHH_gldm_GrayLevelVariance	4.61E-05	0.024031	1.544696	1.2417

wavelet-HHH_gldm_LargeDependenceEmphasis	7.22E-05	0.037629	1.427506	1.224856
wavelet-HHH_gldm_SmallDependenceEmphasis	5.48E-05	0.028527	1.362962	1.469384
wavelet-LLL_firstorder_Mean	6.42E-05	0.033462	1.651712	1.261839
wavelet-LLL_firstorder_Median	3.34E-05	0.017423	1.905058	1.327324
wavelet-LLL_firstorder_Minimum	1.24E-06	0.000647	1.57147	2.581491
wavelet-LLL_firstorder_RootMeanSquared	3.55E-05	0.01848	1.739543	1.290853
wavelet-LLL_gldm_SmallDependenceHighGrayLevelEmphasis	7.62E-05	0.03969	1.40932	1.322063

Table S4. The 2D features with raw p- value, corrected p-value and effect sizes. (grid separation of 1.0 mm).

Feature	P-Value	P-Value-Adjusted	Cohens d 1month day1	Cohens d 3month day1
original_shape_MinorAxisLength	0.000105	0.0312188	1.10099	1.67707808
original_firstorder_Mean	6.49E-05	0.019205	1.643294	1.26509083
original_firstorder_Median	3.75E-05	0.0111128	1.887997	1.32479898
original_firstorder_Minimum	2.28E-08	6.7502E-06	2.452067	3.03546634
original_firstorder_RootMeanSquared	4.09E-05	0.0121079	1.712133	1.27957583
wavelet-LH_firstorder_Maximum	9.41E-05	0.02785728	1.535706	1.38488669
wavelet-LH_firstorder_Minimum	3.99E-05	0.01181156	1.303835	2.27094554
wavelet-LH_firstorder_Range	1.03E-05	0.003062	1.595347	2.09687031
wavelet-LH_glcm_JointAverage	2.47E-05	0.00729983	1.373096	2.40133165
wavelet-LH_glrlm_HighGrayLevelRunEmphasis	1.54E-05	0.00456033	1.351335	2.24710128
wavelet-LH_glrlm_RunPercentage	0.000156	0.04630682	1.429338	1.38394959
wavelet-LH_glrlm_RunVariance	0.000122	0.03600301	1.551018	1.41346822
wavelet-LH_glrlm_ShortRunHighGrayLevelEmphasis	7.57E-06	0.00224081	1.436749	2.3206155
wavelet-LH_glszm_HighGrayLevelZoneEmphasis	1.47E-05	0.00436596	1.355432	2.25942678
wavelet-LH_glszm_SmallAreaHighGrayLevelEmphasis	5.3E-06	0.00156751	1.485718	2.31699861
wavelet-LH_glszm_ZonePercentage	7.99E-05	0.02364446	1.456147	1.4348091
wavelet-LH_gldm_HighGrayLevelEmphasis	1.57E-05	0.0046407	1.349563	2.24296133
wavelet-LH_gldm_SmallDependenceHighGrayLevelEmphasis	8.01E-06	0.00237056	1.92448	1.67740726
wavelet-LH_ngtdm_Complexity	2.26E-05	0.00669969	1.466626	1.58052251
wavelet-HL_glcm_Imc1	2.43E-05	0.00720022	2.618129	1.87262973
wavelet-HL_glcm_Imc2	2.16E-05	0.00640022	1.386282	2.2683739
wavelet-HL_glrlm_LongRunEmphasis	5.62E-06	0.00166378	1.789965	1.53541809
wavelet-HL_glrlm_RunLengthNonUniformityNormalized	7.69E-06	0.00227711	1.717937	1.5389407
wavelet-HL_glrlm_RunPercentage	3.27E-06	0.00096908	1.824188	1.6335517
wavelet-HL_glrlm_RunVariance	3.01E-06	0.00089047	1.866796	1.59810671
wavelet-HL_glrlm_ShortRunEmphasis	1.21E-05	0.00356691	1.683616	1.47769175
wavelet-HL_glszm_ZoneEntropy	1.68E-05	0.00498018	2.115315	1.1173804
wavelet-HL_glszm_ZonePercentage	3.56E-06	0.00105267	1.825886	1.63743692
wavelet-HL_gldm_DependenceVariance	0.000159	0.04704668	1.393817	1.18991299
wavelet-HH_firstorder_10Percentile	2.89E-11	8.5437E-09	3.811114	3.45797132
wavelet-HH_firstorder_90Percentile	3.76E-11	1.1122E-08	3.754319	3.44703005
wavelet-HH_firstorder_Entropy	2.04E-10	6.0395E-08	3.643379	3.08569472
wavelet-HH_firstorder_InterquartileRange	1.13E-12	3.3559E-10	4.680708	4.20839094
wavelet-HH_firstorder_MeanAbsoluteDeviation	3.5E-11	1.0356E-08	3.775319	3.36619598
wavelet-HH_firstorder_Range	0.000119	0.03536327	1.268725	1.35878854
wavelet-HH_firstorder_RobustMeanAbsoluteDeviation	1.19E-12	3.5253E-10	4.576568	4.26880053
wavelet-HH_firstorder_RootMeanSquared	1.27E-09	3.7478E-07	3.146413	2.6236024
wavelet-HH_firstorder_Uniformity	1.42E-10	4.1923E-08	3.920751	3.14877514
wavelet-HH_firstorder_Variance	4.36E-08	1.2908E-05	2.372829	2.12636709
wavelet-HH_glcm_ClusterProminence	0.000143	0.04239513	1.275225	1.18203533
wavelet-HH_glcm_ClusterShade	1.17E-05	0.00345794	1.617692	1.87591489
wavelet-HH_glcm_ClusterTendency	1.71E-08	5.0658E-06	2.49107	2.29222278
wavelet-HH_glcm_Contrast	1.77E-08	5.2277E-06	2.484104	2.28870013

wavelet-HH_glcm_DifferenceAverage	4.24E-11	1.2548E-08	3.706379	3.36100958
wavelet-HH_glcm_DifferenceEntropy	1.85E-10	5.4786E-08	3.668448	3.08334195
wavelet-HH_glcm_DifferenceVariance	1.33E-07	3.9419E-05	2.240324	1.95451644
wavelet-HH_glcm_JointEnergy	3.46E-09	1.0253E-06	3.273951	2.52052592
wavelet-HH_glcm_JointEntropy	4.57E-11	1.3529E-08	4.020356	3.35097314
wavelet-HH_glcm_Imc1	9.64E-06	0.00285487	1.670236	1.79879174
wavelet-HH_glcm_Idm	3.95E-12	1.1685E-09	4.691564	3.85835007
wavelet-HH_glcm_Id	4.66E-12	1.3802E-09	4.62996	3.82521381
wavelet-HH_glcm_InverseVariance	1.44E-10	4.2527E-08	3.307412	3.22185061
wavelet-HH_glcm_MaximumProbability	5.02E-12	1.4863E-09	4.807898	3.93097651
wavelet-HH_glcm_SumEntropy	9.75E-11	2.8849E-08	3.834048	3.2044193
wavelet-HH_glcm_SumSquares	1.74E-08	5.1467E-06	2.487555	2.29044681
wavelet-HH_glrlm_GrayLevelNonUniformityNormalized	4.33E-09	1.2812E-06	3.134446	2.6420227
wavelet-HH_glrlm_GrayLevelVariance	1.55E-07	4.5819E-05	2.213021	1.93812111
wavelet-HH_glrlm_LongRunEmphasis	8.81E-08	2.6089E-05	2.716241	2.12781134
wavelet-HH_glrlm_RunLengthNonUniformityNormalized	5.65E-12	1.6735E-09	4.777386	3.84971318
wavelet-HH_glrlm_RunPercentage	4.79E-11	1.4182E-08	4.191069	3.45329217
wavelet-HH_glrlm_RunVariance	5.26E-08	1.5563E-05	2.866623	2.1231807
wavelet-HH_glrlm_ShortRunEmphasis	1.2E-09	3.5588E-07	3.415948	2.91222189
wavelet-HH_glszm_GrayLevelNonUniformityNormalized	9.35E-07	0.0002769	2.162898	2.0805765
wavelet-HH_glszm_GrayLevelVariance	1.69E-05	0.00501367	1.710343	1.31215968
wavelet-HH_glszm_SizeZoneNonUniformityNormalized	2.84E-07	8.4164E-05	1.952183	2.39521986
wavelet-HH_glszm_SmallAreaEmphasis	1.26E-06	0.00037256	1.733484	2.25923799
wavelet-HH_glszm_ZonePercentage	2.05E-13	6.0578E-11	5.166494	4.85534491
wavelet-HH_gldm_DependenceVariance	5.35E-07	0.00015829	3.423621	2.08254299
wavelet-HH_gldm_GrayLevelVariance	1.16E-07	3.4419E-05	2.215268	2.00155775
wavelet-HH_gldm_LargeDependenceEmphasis	1.64E-07	4.8449E-05	2.413458	1.86444407
wavelet-HH_gldm_SmallDependenceEmphasis	7.99E-07	0.00023648	2.087334	1.75188483
wavelet-HH_gldm_SmallDependenceHighGrayLevelEmphasis	0.000131	0.03888052	1.23144	1.24856595
wavelet-HH_ngtdm_Complexity	3.31E-05	0.00978765	1.432534	1.37071622
wavelet-HH_ngtdm_Contrast	1.61E-05	0.00475779	1.759386	1.83135914
wavelet-LL_firstorder_Mean	6.46E-05	0.01913189	1.644632	1.26363567
wavelet-LL_firstorder_Median	3.33E-05	0.00985055	1.898041	1.32824822
wavelet-LL_firstorder_Minimum	5.25E-06	0.00155472	1.467239	2.45313141
wavelet-LL_firstorder_RootMeanSquared	3.68E-05	0.01088337	1.732184	1.28880616
wavelet-LL_gldm_SmallDependenceHighGrayLevelEmphasis	8.63E-05	0.02555523	1.430532	1.27460602

Table S5. The 3D features with raw p- value, corrected p-value and effect sizes. (grid separation of 1.0 mm).

Feature	P-Value	P-Value-Adjusted	Cohens d 1month day1	Cohens d 3month day1
original_shape_MinorAxisLength	8.975E-05	0.0489151	1.115676074	1.697238382
original_firstorder_Mean	6.664E-05	0.0363192	1.650146726	1.260587301
original_firstorder_Median	3.637E-05	0.0198238	1.897883744	1.325584276
original_firstorder_Minimum	3.207E-08	1.748E-05	2.755337423	2.609099159
original_firstorder_RootMeanSquared	4.157E-05	0.0226579	1.714966358	1.276507073
wavelet-LLH_firstorder_90Percentile	9.761E-06	0.0053198	2.793300892	1.605522717
wavelet-LLH_firstorder_InterquartileRange	1.125E-05	0.0061297	2.220657264	1.550822719
wavelet-LLH_firstorder_MeanAbsoluteDeviation	3.066E-05	0.0167081	2.646807152	1.471084431
wavelet-LLH_firstorder_RobustMeanAbsoluteDeviation	1.305E-05	0.0071138	2.320136482	1.51464261
wavelet-LLH_firstorder_Variance	5.038E-05	0.027456	2.602397607	1.420956514
wavelet-LLH_glcm_ClusterTendency	7.892E-05	0.0430127	2.34372653	1.404311657
wavelet-LLH_glcm_Contrast	4.252E-05	0.0231722	2.719628476	1.491662578
wavelet-LLH_glcm_Correlation	8.078E-05	0.0440246	1.585912872	2.028135921
wavelet-LLH_glcm_DifferenceAverage	4.537E-05	0.0247262	2.63092022	1.49111439
wavelet-LLH_glcm_DifferenceVariance	8.889E-05	0.0484446	2.099944466	1.519181089
wavelet-LLH_glcm_JointEntropy	5.683E-05	0.0309717	2.286646856	1.485542538
wavelet-LLH_glcm_InverseVariance	7.946E-05	0.0433073	2.201866563	1.424606297
wavelet-LLH_glcm_SumSquares	5.705E-05	0.0310917	2.535733247	1.448575498
wavelet-LLH_glrlm_RunLengthNonUniformityNormalized	5.83E-06	0.0031772	2.339497413	1.807051428

wavelet-LLH_glrlm_RunPercentage	7.781E-06	0.0042408	2.053798494	1.830229465
wavelet-LLH_glrlm_ShortRunEmphasis	1.546E-05	0.008426	2.02183838	1.751578832
wavelet-LLH_glszm_ZonePercentage	3.018E-06	0.0016447	3.685094877	1.665458436
wavelet-LLH_gldm_GrayLevelVariance	5.268E-05	0.0287081	2.586122718	1.417075349
wavelet-LLH_gldm_SmallDependenceEmphasis	8.334E-05	0.0454209	1.733151335	1.082792458
wavelet-LHL_firstorder_Minimum	8.866E-05	0.0483182	1.21699064	2.013214026
wavelet-LHL_firstorder_Range	3.313E-05	0.0180562	1.474763256	1.7721945
wavelet-LHL_glcm_JointAverage	7.951E-05	0.0433324	1.237515957	2.008854088
wavelet-LHL_glrlm_HighGrayLevelRunEmphasis	5.497E-05	0.029958	1.210009729	1.966970111
wavelet-LHL_glrlm_RunPercentage	7.391E-05	0.0402832	1.628428119	1.455066948
wavelet-LHL_glrlm_ShortRunHighGrayLevelEm-phasis	3.4E-05	0.0185294	1.263514496	2.026544162
wavelet-LHL_glszm_HighGrayLevelZoneEmphasis	4.945E-05	0.0269512	1.218853022	1.989073532
wavelet-LHL_glszm_SmallAreaHighGrayLevelEm-phasis	1.564E-05	0.0085216	1.351408683	2.119557543
wavelet-LHL_gldm_HighGrayLevelEmphasis	5.546E-05	0.0302257	1.20912546	1.965508178
wavelet-LHL_gldm_SmallDepend-enceHighGrayLevelEmphasis	3.164E-06	0.0017246	2.26106364	1.606612241
wavelet-LHL_ngtdm_Complexity	3.511E-05	0.0191326	1.398856701	1.558783254
wavelet-LHH_firstorder_10Percentile	8.566E-10	4.669E-07	4.142815163	2.86030337
wavelet-LHH_firstorder_90Percentile	5.483E-09	2.988E-06	3.817520486	2.757053978
wavelet-LHH_firstorder_Entropy	7.676E-09	4.183E-06	3.207031969	2.801454406
wavelet-LHH_firstorder_InterquartileRange	7.373E-11	4.018E-08	5.055487158	3.249259194
wavelet-LHH_firstorder_MeanAbsoluteDeviation	3.118E-09	1.699E-06	3.920907092	2.655545879
wavelet-LHH_firstorder_Median	7.125E-06	0.003883	1.637125624	1.552871751
wavelet-LHH_firstorder_Minimum	6.947E-05	0.037862	1.403604505	1.350134013
wavelet-LHH_firstorder_RobustMeanAbso-luteDeviation	9.851E-11	5.369E-08	4.957830328	3.241700688
wavelet-LHH_firstorder_RootMeanSquared	1.207E-07	6.58E-05	2.909901988	2.100271201
wavelet-LHH_firstorder_Uniformity	1.312E-09	7.149E-07	3.358950079	3.377569964
wavelet-LHH_firstorder_Variance	2.857E-06	0.001557	2.308822511	1.528925184
wavelet-LHH_glcm_JointAverage	7.187E-05	0.0391695	1.38319902	1.383499313
wavelet-LHH_glcm_ClusterTendency	6.01E-07	0.0003276	2.706355788	1.764290234
wavelet-LHH_glcm_Contrast	5.938E-07	0.0003236	2.712672289	1.765206729
wavelet-LHH_glcm_DifferenceAverage	6.218E-09	3.389E-06	3.858164911	2.631223405
wavelet-LHH_glcm_DifferenceEntropy	3.584E-08	1.953E-05	2.715450507	2.73604622
wavelet-LHH_glcm_DifferenceVariance	2.427E-06	0.0013225	2.344903868	1.630703795
wavelet-LHH_glcm_JointEnergy	1.009E-08	5.497E-06	2.624358713	3.385937694
wavelet-LHH_glcm_JointEntropy	6.787E-09	3.699E-06	3.077186405	3.062647895
wavelet-LHH_glcm_Idm	9.515E-10	5.186E-07	3.694707805	3.486304085
wavelet-LHH_glcm_Id	9.693E-10	5.283E-07	3.673202531	3.492771784
wavelet-LHH_glcm_InverseVariance	3.036E-05	0.0165468	1.85751884	1.323045485
wavelet-LHH_glcm_MaximumProbability	9.536E-10	5.197E-07	3.591302372	3.442001424
wavelet-LHH_glcm_SumEntropy	2.021E-08	1.101E-05	2.829391505	2.88363452
wavelet-LHH_glcm_SumSquares	5.974E-07	0.0003256	2.709525575	1.7647506
wavelet-LHH_glrlm_GrayLevelNonUniformi-tyNormalized	5.914E-08	3.223E-05	2.465933706	2.660561857
wavelet-LHH_glrlm_GrayLevelVariance	5.472E-06	0.0029821	2.213502451	1.477472638
wavelet-LHH_glrlm_LongRunEmphasis	1.955E-07	0.0001066	2.112370945	2.397990986
wavelet-LHH_glrlm_RunLengthNonUniformi-tyNormalized	2.288E-10	1.247E-07	3.364955226	3.924158923
wavelet-LHH_glrlm_RunPercentage	5.37E-10	2.926E-07	3.086312678	3.767120693
wavelet-LHH_glrlm_RunVariance	3.584E-08	1.953E-05	2.400476158	2.651842835
wavelet-LHH_glrlm_ShortRunEmphasis	1.267E-08	6.907E-06	2.530998954	2.985714582
wavelet-LHH_glszm_ZonePercentage	5.774E-08	3.147E-05	2.955448014	2.180703553
wavelet-LHH_gldm_GrayLevelVariance	4.35E-06	0.0023708	2.201350512	1.485667023
wavelet-LHH_gldm_LargeDependenceEmphasis	1.483E-05	0.0080811	1.581407449	1.527333872
wavelet-LHH_gldm_SmallDepend-enceHighGrayLevelEmphasis	6.601E-05	0.0359773	1.561618413	1.205085839
wavelet-HLL_glrlm_LongRunEmphasis	1.418E-05	0.0077271	1.53366236	1.86242258
wavelet-HLL_glrlm_RunLengthNonUniformi-tyNormalized	4.927E-06	0.0026852	1.666791115	1.740027751
wavelet-HLL_glrlm_RunPercentage	3.913E-06	0.0021328	1.67872548	1.891482441

wavelet-HLL_glrlm_RunVariance	2.256E-05	0.0122976	1.497120217	1.942524355
wavelet-HLL_glrlm_ShortRunEmphasis	7.348E-06	0.0040044	1.622837867	1.694673863
wavelet-HLL_glszm_ZonePercentage	1.832E-05	0.0099855	1.620676954	1.424403472
wavelet-HLH_firstorder_10Percentile	3.035E-07	0.0001654	2.635348489	1.686731063
wavelet-HLH_firstorder_90Percentile	2.879E-07	0.0001569	2.64129858	1.713180993
wavelet-HLH_firstorder_Entropy	3.536E-06	0.0019269	2.298954323	1.48138804
wavelet-HLH_firstorder_InterquartileRange	2.902E-08	1.582E-05	2.758821748	2.037741059
wavelet-HLH_firstorder_MeanAbsoluteDeviation	4.484E-07	0.0002444	2.501287107	1.677166113
wavelet-HLH_firstorder_Median	4.339E-08	2.365E-05	2.236732157	2.347458407
wavelet-HLH_firstorder_Minimum	1.703E-05	0.0092793	1.645127579	1.694494304
wavelet-HLH_firstorder_RobustMeanAbsoluteDeviation	3.444E-08	1.877E-05	2.784859809	1.999431045
wavelet-HLH_firstorder_RootMeanSquared	5.676E-06	0.0030933	2.146526831	1.447352544
wavelet-HLH_firstorder_Uniformity	1.485E-06	0.0008095	2.410685991	1.557025308
wavelet-HLH_firstorder_Variance	1.41E-05	0.0076852	1.843165197	1.330413166
wavelet-HLH_glcm_JointAverage	1.196E-05	0.006518	1.609964233	1.777601084
wavelet-HLH_glcm_ClusterShade	1.114E-07	6.069E-05	2.127481668	2.406647177
wavelet-HLH_glcm_ClusterTendency	2.736E-05	0.0149133	1.734676366	1.268196318
wavelet-HLH_glcm_Contrast	2.686E-05	0.0146384	1.738560269	1.268635722
wavelet-HLH_glcm_DifferenceAverage	4.48E-06	0.0024419	2.11472298	1.435244288
wavelet-HLH_glcm_DifferenceEntropy	3.596E-06	0.00196	2.389429724	1.476051743
wavelet-HLH_glcm_DifferenceVariance	4.734E-05	0.0257985	1.687522946	1.215741683
wavelet-HLH_glcm_JointEnergy	4.563E-07	0.0002487	2.788441836	1.695505561
wavelet-HLH_glcm_JointEntropy	1.917E-06	0.0010449	2.447787169	1.538951706
wavelet-HLH_glcm_Idm	1.159E-06	0.0006314	2.433763903	1.574730746
wavelet-HLH_glcm_Id	1.004E-06	0.0005471	2.46641793	1.589691127
wavelet-HLH_glcm_MaximumProbability	1.271E-05	0.0069267	2.057766402	1.30950707
wavelet-HLH_glcm_SumEntropy	2.655E-06	0.0014471	2.424284464	1.505288212
wavelet-HLH_glcm_SumSquares	2.711E-05	0.0147747	1.736626019	1.268416656
wavelet-HLH_glrlm_GrayLevelNonUniformityNormalized	2.206E-06	0.0012025	2.443231988	1.548306845
wavelet-HLH_glrlm_GrayLevelVariance	5.073E-05	0.0276483	1.681080754	1.224269901
wavelet-HLH_glrlm_HighGrayLevelRunEmphasis	7.032E-05	0.0383242	1.306240471	1.384716107
wavelet-HLH_glrlm_LongRunEmphasis	3.096E-06	0.0016873	2.027584755	1.622396308
wavelet-HLH_glrlm_RunLengthNonUniformityNormalized	2.073E-08	1.13E-05	3.32605465	2.061733648
wavelet-HLH_glrlm_RunPercentage	2.53E-08	1.379E-05	3.159476446	2.069118598
wavelet-HLH_glrlm_RunVariance	3.155E-06	0.0017194	2.058771818	1.565875051
wavelet-HLH_glrlm_ShortRunEmphasis	8.908E-08	4.855E-05	2.907897844	1.971844828
wavelet-HLH_glrlm_ShortRunHighGrayLevelEmphasis	4.727E-05	0.0257633	1.363850263	1.388038583
wavelet-HLH_glszm_HighGrayLevelZoneEmphasis	8.026E-05	0.0437428	1.303004763	1.352576556
wavelet-HLH_glszm_SmallAreaHighGrayLevelEmphasis	6.742E-05	0.0367452	1.337095877	1.34663409
wavelet-HLH_glszm_ZonePercentage	5.426E-09	2.957E-06	3.455538372	2.263292345
wavelet-HLH_gldm_GrayLevelVariance	3.832E-05	0.0208859	1.671830577	1.244259112
wavelet-HLH_gldm_HighGrayLevelEmphasis	6.77E-05	0.0368984	1.308997182	1.389860241
wavelet-HLH_gldm_SmallDependenceEmphasis	1.393E-06	0.0007594	2.266525714	1.599865284
wavelet-HLH_gldm_SmallDependenceHighGrayLevelEmphasis	4.257E-06	0.00232	1.892847656	1.532119215
wavelet-HHL_firstorder_10Percentile	2.294E-11	1.25E-08	3.78641564	3.56778685
wavelet-HHL_firstorder_90Percentile	3.432E-11	1.87E-08	3.71536903	3.512390819
wavelet-HHL_firstorder_Entropy	3.926E-10	2.14E-07	3.506864497	3.005285983
wavelet-HHL_firstorder_InterquartileRange	9.833E-13	5.359E-10	4.676217591	4.324682468
wavelet-HHL_firstorder_MeanAbsoluteDeviation	2.82E-11	1.537E-08	3.776147733	3.459693227
wavelet-HHL_firstorder_RobustMeanAbsoluteDeviation	1.164E-12	6.345E-10	4.558797234	4.328608199
wavelet-HHL_firstorder_RootMeanSquared	8.854E-10	4.825E-07	3.16144713	2.716245718
wavelet-HHL_firstorder_Uniformity	2.142E-08	1.168E-05	2.838917104	2.320722674
wavelet-HHL_firstorder_Variance	3.342E-08	1.821E-05	2.386610166	2.184978016
wavelet-HHL_glcm_ClusterShade	4.64E-06	0.0025288	1.618543898	1.972059676
wavelet-HHL_glcm_ClusterTendency	3.961E-09	2.159E-06	2.717055567	2.524841334

wavelet-HHL_glcm_Contrast	3.887E-09	2.118E-06	2.717769477	2.530183749
wavelet-HHL_glcm_DifferenceAverage	1.277E-11	6.961E-09	3.99871889	3.61997741
wavelet-HHL_glcm_DifferenceEntropy	3.327E-10	1.813E-07	3.498603906	3.04023671
wavelet-HHL_glcm_DifferenceVariance	5.826E-08	3.175E-05	2.351883024	2.073660123
wavelet-HHL_glcm_JointEnergy	3.649E-06	0.0019888	1.985896623	1.648794752
wavelet-HHL_glcm_JointEntropy	1.47E-10	8.013E-08	3.730844043	3.150730477
wavelet-HHL_glcm_Idm	4.641E-10	2.529E-07	3.604637349	2.877179195
wavelet-HHL_glcm_Id	2.052E-10	1.118E-07	3.756989437	3.03532936
wavelet-HHL_glcm_InverseVariance	1.368E-11	7.456E-09	4.449557643	3.565250895
wavelet-HHL_glcm_MaximumProbability	1.637E-08	8.921E-06	2.868614751	2.337576069
wavelet-HHL_glcm_SumEntropy	3.697E-10	2.015E-07	3.487535829	3.014585928
wavelet-HHL_glcm_SumSquares	3.924E-09	2.138E-06	2.717414087	2.527516104
wavelet-HHL_glrlm_GrayLevelNonUniformi-tyNormalized	3.176E-08	1.731E-05	2.759727555	2.344029082
wavelet-HHL_glrlm_GrayLevelVariance	5.435E-08	2.962E-05	2.335557565	2.101784054
wavelet-HHL_glrlm_LongRunEmphasis	2.409E-06	0.0013129	2.006475305	1.707337309
wavelet-HHL_glrlm_RunEntropy	6.898E-08	3.759E-05	2.483756806	2.174340938
wavelet-HHL_glrlm_RunLengthNonUniformi-tyNormalized	8.287E-10	4.516E-07	3.484803464	2.816088626
wavelet-HHL_glrlm_RunPercentage	4.355E-09	2.373E-06	3.172220927	2.501404542
wavelet-HHL_glrlm_RunVariance	2.487E-06	0.0013555	2.009577013	1.627947871
wavelet-HHL_glrlm_ShortRunEmphasis	1.467E-08	7.994E-06	2.882479657	2.405961898
wavelet-HHL_glszm_GrayLevelVariance	4.95E-06	0.0026978	1.906670987	1.452797546
wavelet-HHL_glszm_SizeZoneNonUniformi-tyNormalized	1.047E-08	5.707E-06	2.577501473	2.905840166
wavelet-HHL_glszm_SmallAreaEmphasis	1.034E-07	5.638E-05	2.211883964	2.64252374
wavelet-HHL_glszm_ZonePercentage	7.652E-13	4.17E-10	4.659198266	4.311573605
wavelet-HHL_gldm_DependenceNonUniformi-tyNormalized	8.454E-10	4.608E-07	3.444093138	3.335145228
wavelet-HHL_gldm_DependenceVariance	5.141E-06	0.0028021	2.200796607	1.362481678
wavelet-HHL_gldm_GrayLevelVariance	3.347E-08	1.824E-05	2.387672215	2.184101665
wavelet-HHL_gldm_LargeDependenceEmphasis	1.778E-05	0.0096879	1.740916944	1.267869235
wavelet-HHL_gldm_SmallDependenceEmphasis	4.875E-08	2.657E-05	2.367783749	2.173089063
wavelet-HHL_gldm_SmallDepend-enceHighGrayLevelEmphasis	3.045E-05	0.0165934	1.374028882	1.43583961
wavelet-HHL_ngtdm_Complexity	1.646E-05	0.0089695	1.510775181	1.444242285
wavelet-HHL_ngtdm_Contrast	1.398E-06	0.0007621	1.784657287	1.987975801
wavelet-HHH_firstorder_10Percentile	2.132E-10	1.162E-07	4.058043406	2.826455098
wavelet-HHH_firstorder_90Percentile	7.009E-10	3.82E-07	3.773049508	2.597162834
wavelet-HHH_firstorder_Entropy	1.416E-05	0.0077161	1.885353497	1.344718461
wavelet-HHH_firstorder_InterquartileRange	3.764E-12	2.052E-09	4.855643752	3.619508638
wavelet-HHH_firstorder_MeanAbsoluteDevia-tion	1.82E-09	9.921E-07	3.553545372	2.434001206
wavelet-HHH_firstorder_Median	1.943E-06	0.0010591	1.839849416	1.776250144
wavelet-HHH_firstorder_RobustMeanAbso-luteDeviation	9.654E-12	5.261E-09	4.674456027	3.390407329
wavelet-HHH_firstorder_RootMeanSquared	2.701E-07	0.0001472	2.625099294	1.773066293
wavelet-HHH_firstorder_Uniformity	2.24E-05	0.0122093	1.728298081	1.308500586
wavelet-HHH_firstorder_Variance	4.648E-06	0.0025329	1.91352507	1.461966281
wavelet-HHH_glcm_ClusterShade	7.967E-06	0.0043419	2.862489924	2.01866333
wavelet-HHH_glcm_DifferenceAverage	3.643E-05	0.0198533	1.603922559	1.277193086
wavelet-HHH_glcm_DifferenceEntropy	6.83E-06	0.0037224	2.013022519	1.442980758
wavelet-HHH_glcm_JointEnergy	5.509E-06	0.0030023	1.902011556	1.486950437
wavelet-HHH_glcm_JointEntropy	5.818E-06	0.0031707	1.975228113	1.466407479
wavelet-HHH_glcm_Idm	1.423E-05	0.007756	1.728602805	1.382551324
wavelet-HHH_glcm_Id	1.264E-05	0.0068868	1.744508036	1.395994962
wavelet-HHH_glcm_SumEntropy	6.868E-06	0.0037429	1.979835935	1.445382658
wavelet-HHH_glrlm_GrayLevelNonUniformi-tyNormalized	7.1E-06	0.0038697	1.971202044	1.417531263
wavelet-HHH_glrlm_LongRunEmphasis	3.607E-05	0.0196588	1.514405896	1.362695608
wavelet-HHH_glrlm_RunLengthNonUniformi-tyNormalized	7.82E-06	0.0042617	1.821969472	1.451358661
wavelet-HHH_glrlm_RunPercentage	4.656E-06	0.0025375	1.846105513	1.551716238

wavelet-HHH_glrlm_ShortRunEmphasis	2.571E-06	0.0014012	2.012795423	1.591830914
wavelet-HHH_glszm_ZonePercentage	2.654E-08	1.447E-05	2.631810369	2.146877163
wavelet-HHH_gldm_SmallDependenceEmphasis	1.376E-06	0.0007498	2.253548671	1.760945443
wavelet-HHH_gldm_SmallDependenceHighGrayLevelEmphasis	1.152E-06	0.0006279	1.816833819	1.845912924
wavelet-LLL_firstorder_Mean	6.628E-05	0.0361208	1.65181094	1.259357134
wavelet-LLL_firstorder_Median	3.306E-05	0.0180185	1.905104183	1.326889582
wavelet-LLL_firstorder_Minimum	1.595E-06	0.0008691	1.54278643	2.612617362
wavelet-LLL_firstorder_RootMeanSquared	3.704E-05	0.0201866	1.736245969	1.286735997
wavelet-LLL_gldm_SmallDependenceHighGrayLevelEmphasis	5.986E-05	0.0326264	1.386581521	1.367039056

Table S6. The 3 significant features (2D) for both resampled grid distance of 0.5 mm and 1.0 mm.

Patient	Grid resample distance 0.5 mm								
	Mean Absolute deviation (A.U)			HH wavelet filtered 90th Percentile (A.U)			HH wavelet filtered Interquartile Range (A.U)		
	Day 1	1 month	3 months	Day 1	1 month	3 months	Day 1	1 month	3 months
P0	1.45	0.38	0.54	3.65	1.14	1.38	3.43	0.84	1.27
P1	1.17	0.22	0.19	2.93	0.6	0.52	2.77	0.51	0.44
P2	1.08	0.29	0.39	2.56	0.79	1.03	2.6	0.66	0.9
P3	0.89	0.23	0.26	2.26	0.66	0.72	2.1	0.53	0.59
P4	1.04	0.28	0.3	2.55	0.88	0.85	2.47	0.63	0.68
P5	1.01	0.37	0.45	2.84	0.94	1.23	2.57	0.86	1.1
P6	1.28	0.17	0.26	2.57	0.45	0.72	2.41	0.41	0.61
P7	1.06	0.28	0.36	2.53	0.82	1.06	2.54	0.65	0.85
P8	1.3	0.39	0.41	3.08	1.01	1.07	3.08	0.91	0.95
P9	1.34	0.21	0.25	3.19	0.54	0.72	3.2	0.48	0.55

Patient	Grid resample distance 1.0 mm								
	HH wavelet filtered GLSZM Zone percentage (A.U)			Run length non-uniformity(A.U)			Mean absolute Deviation (A.U)		
	Day 1	1 month	3 months	Day 1	1 month	3 months	Day 1	1 month	3 months
P0	0.55	0.22	0.27	2837.47	658.59	476.65	8.36	2.84	4.09
P1	0.44	0.067	0.05	58089.55	5377.18	4191.13	6.64	1.89	1.7
P2	0.38	0.11	0.14	4721.71	771.19	801.83	5.96	2.41	2.97
P3	0.35	0.09	0.1	144338.7	33013.92	36374.23	5.35	2.12	2.32
P4	0.42	0.14	0.13	38990.76	8151.68	2291.33	6.16	2.19	2.44
P5	0.45	0.17	0.22	13428.11	3170.96	4143.14	6.51	3.41	3.9
P6	0.46	0.05	0.14	2246.9	613.4	950.67	6.62	1.67	2.53
P7	0.41	0.12	0.18	41069.59	17016.91	10649.25	6.13	1.54	3.14
P8	0.48	0.19	0.22	28129.67	1660.72	1657.99	7.4	3.26	3.55
P9	0.49	0.05	0.11	163032.9	18638.79	19537.71	7.65	1.98	2.4

Table S7. The 3 significant features (3D) for both resampled grid distance of 0.5 mm and 1.0 mm.

Patient	Grid resample distance 0.5 mm/Grid resample distance 1.0 mm								
	HHH wavelet filtered Interquartile Range (A.U)			HHL wavelet filtered Interquartile Range (A.U)			HHL wavelet filtered Robust Mean Absolute deviation (A.U)		
	Day 1	1 month	3 months	Day 1	1 month	3 months	Day 1	1 month	3 months
P0	3.69	1.32	1.48	27.58	9.15	13.61	11.65	3.92	5.75
P1	3.07	0.87	0.72	21.88	6.23	5.67	9.26	2.63	2.39
P2	0.19	0.05	0.072	3.63	0.93	1.25	1.51	0.41	0.54
P3	2.31	0.72	0.8	17.55	6.88	7.51	7.47	2.96	3.25
P4	2.44	1.06	1.04	20.39	7.14	8.17	8.6	3.07	3.45
P5	2.93	1.25	1.46	21.38	11.4	12.84	9.06	4.81	5.41
P6	2.51	0.57	0.81	21.59	5.57	8.37	9.24	2.33	3.54
P7	2.53	1.05	1.41	20.53	8.23	10.41	8.57	3.54	4.41
P8	3.06	1.11	1.09	24.64	10.81	11.77	10.36	4.58	4.98
P9	3.14	0.61	0.73	25.35	6.53	7.54	10.69	2.77	3.35

Note- The significant features and their values are consistent for 3D features over the resampled grid distances.
